# Ginsenoside Rg3 Promotes Cell Growth Through Activation of mTORC1

**DOI:** 10.3389/fcell.2021.730309

**Published:** 2021-09-13

**Authors:** Wei Liu, Sheng-Xiong Zhang, Bo Ai, Hua-Feng Pan, Dan Zhang, Yu Jiang, Lei-Hao Hu, Ling-Ling Sun, Zhe-Sheng Chen, Li-Zhu Lin

**Affiliations:** ^1^Lingnan Medical Research Center, Guangzhou University of Chinese Medicine, Guangzhou, China; ^2^Integrative Cancer Centre, The First Affiliated Hospital of Guangzhou, University of Chinese Medicine, Guangzhou, China; ^3^Guangdong Work Injury Rehabilitation Hospital, Guangzhou, China; ^4^Department of Thoracic Surgery, Tongji Medical College, Huazhong University of Science and Technology, Wuhan, China; ^5^Department of Pharmacology and Chemical Biology, University of Pittsburgh School of Medicine, Pittsburgh, PA, United States; ^6^Institute for Biotechnology, St. John’s University, Queens, NY, United States

**Keywords:** ginsenoside Rg3, mTORC1, cell growth, cancer, *Panax ginseng*

## Abstract

Ginsenoside Rg3 is a steroidal saponin isolated from *Panax ginseng.* Previous studies have shown that Rg3 treatment downregulates the activity of rapamycin complex 1 (mTORC1) activity and inhibits the growth of cancer cells. However, the inhibitory effect of Rg3 on cancer cells is associated with high concentrations of Rg3 that are difficult to achieve *in vivo*. The human cervix adenocarcinoma HeLa cells were treated with Rg3. The protein levels of AMP-activated protein kinase alpha (AMPKα), protein kinase B(Akt), ribosomal S6 protein(S6), and Erk were determined by immunoblotting analyses. We used a fluorescent probe to detect reactive oxygen species (ROS) production in living cells. The oxygen consumption rate (OCR) was examined by the Seahorse Extracellular Flux Analyzer. The content of adenosine triphosphate (ATP) was measured by ATPlite kit and Mitotracker was applied to detect the mitochondria. We showed that at lower concentrations, Rg3 activates mTORC1 independent of AKT and AMP-activated protein kinase (AMPK). Rg3 promotes mitochondrial biogenesis and function, increases the oxygen consumption of mitochondria and the content of ATP. This effect is in contrast to that of high concentrations of Rg3, which inhibits cell growth. These findings demonstrate a pro-growth activity of Rg3 that acts through mTORC1 and mitochondrial biogenesis and suggest a dose-dependent effect of Rg3 on tumor cell growth.

## Graphical Abstract

Ginsenoside Rg3 promotes cell growth through activation of mTORC1 and increasing mitochondrial biogenesis.

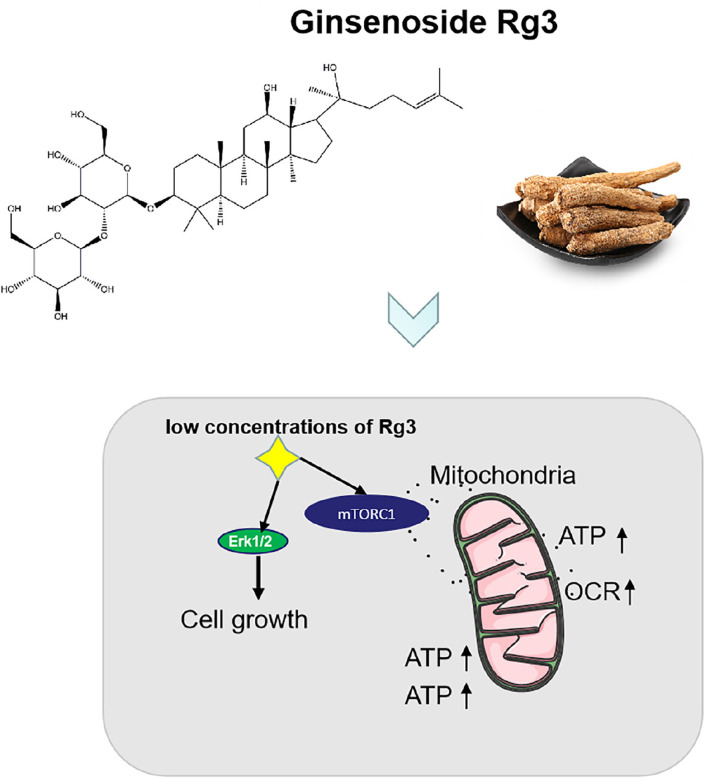

## Introduction

Rg3 is one of the pharmaceutical ingredients extracted from *Panax ginseng*. Its preparations have been widely used in cancer treatment to enhance patient’s general health and improve the efficacy of chemotherapeutic agents (Lu et al., 2008; Zhou et al., 2016; Guo et al., 2018; Pan et al., 2019; Wang et al., 2019). A national survey on men and women in the United States estimated that 4–5% of those aged 45–64 years used ginseng (Kaufman et al., 2002). Research to better understanding the therapeutic potential of Asian ginseng has been supported by the National Center for Complementary and alternative medicine (Jia et al., 2009).

Previous studies have shown that Rg3 possesses activities to inhibit cell growth and induce apoptosis. It has been shown that Rg3 induces apoptosis *via* classical mitochondria-dependent caspase activation (Kim et al., 2013) and the death receptor-dependent extrinsic pathway (Kim et al., 2014). Rg3 exerts cytotoxic effects by activating the p53 signaling pathway and subsequently inducing apoptosis (Yuan et al., 2010; Zhang et al., 2015). In addition, Rg3 has been shown to inhibit mutant p53 and NF-κB signaling, possibly *via* the inactivation of extracellular signal-regulated kinase (ERK) and AKT to activate the mitochondrial cell death pathway (Kim et al., 2013, 2014; Aziz et al., 2016). Experiments studies also suggest that Rg3 *in vivo* and *vitro* can inhibit the growth of a variety of tumor cells both *in vivo* and *vitro* (Wang et al., 2014, 2018; Yuan et al., 2017; Chen et al., 2019). Previous studies have proposed that Rg3 inhibits cell growth by downregulation of lncRNA CCAT1 (Li and Qi, 2019), inhibition of Wnt/β-Catenin, NF-κB, and mitogen-activated protein kinases (MAPK)/ERK signaling pathways (Kim et al., 2004; Lee et al., 2009; Yuan et al., 2010; He et al., 2011; Joo et al., 2015; Yang et al., 2016, 2017). These activities of Rg3 are believed to underlie the enhanced chemotherapeutic efficacy in clinical studies (Lu et al., 2008; Zhou et al., 2016) and in experimental mice (Chang et al., 2014; Shi et al., 2020). However, the pro-apoptosis and anti-proliferation effects of Rg3 occur only at high concentrations (>100 mM), which can be difficult to achieve *in vivo* due the low bioavailability of Rg3 (Xie et al., 2005; Jia et al., 2009).

In the present study we examined the effects of Rg3 on cell signaling and proliferation at low concentrations. We show that at at low concentrations, Rg3 activates the mTORC1 and promotes cell growth. This observation provides information on the efficacy of Rg3 and guide the selection of a safe dose for further study or human use as a certain *in vivo* concentration cannot be guaranteed.

## Materials and Methods

### Cell Line and Culture

The human cervix adenocarcinoma HeLa cells obtained from the American Type Culture Collection (ATCC, Manassas, VA, United States) were cultured in EMEM supplemented with 10% of fetal bovine serum (FBS) (Sigma-Aldrich Chemical Co., St. Louis, MO, United States). In order to inhibit the activity of mTORC1 and induce the baseline level of PS6 in HeLa cells, serum starved cells were cultured in serum starvation condition (0.5% FBS) for 24 h after cells reaching confluence. The cell cultures were incubated at 37°C in a humidified atmosphere with 5% CO_2_. The cell cultures were incubated at 37°C in a humidified atmosphere with 5% CO_2_.

### Cell Counting Kit-8 (CCK8 Assay)

Cell growth was assessed using the Cell Counting Kit-8 (CCK8) assay. CCK8 assay was conducted in accordance with the manufacturer’s instructions (BS350B, Biosharp, China). Briefly, HeLa cells (1 × 10^3^ cells/well) were seeded in 96-well plates. The next day, the cells were treated with Rg3 at various doses. After 24 h, 10 μl of CCK8 solution was added to each well and incubated for 1 h before the light absorbance was measured at 450 nm.

### Antibodies and Reagents

The primary antibodies used in this study include the following: mouse anti-AMPKα (Cat. # 2793), rabbit anti-phospho-AMPKα (T172) (Cat. # 2535), rabbit anti-Akt (Cat. # 9272), rabbit anti-phospho-Akt (Thr-308) (Cat. # 4056), rabbit anti-phospho-Akt (S473) (Cat. # 4060), mouse anti-S6 (Cat. # 2317), phospho-S6(S235/236) (Cat. # 2211), anti-Erk (Cat. # 4695S), and anti-phospho-Erk (Cat. # 9101S) were purchased from Cell Signaling Technology, mouse anti-β-actin (Cat. # 612657) was purchased from BD Transduction Laboratories. Rg3 (Cat. #SML0184, Sigma, purity >98%) was dissolved in dimethyl sulfoxide (DMSO), The N-acetyl-l-cysteine (NAC) (Cat. #194603, MP Biomedical) was dissolved in H_2_O, Oligomycin (Cat. # SLBZ3164, Sigma) and FCCP (Cat. #C2920, Sigma), and tert-Butyl hydroperoxide (Cat. #180340050, ACROS Organics).

### Western Blot Analysis

Cells were lysed with lysis buffer. Samples were subjected to sodium dodecyl sulfate polyacrylamide gel (SDS-PAGE) electrophoresis. Western blot analysis was performed as described previously (Zhong et al., 2016) and the immobilized proteins were visualized by the enhanced chemiluminescent (ECL) detection system. Antibody concentrations were optimized with various dilutions to ensure that the blotting signals are linear to the levels of loaded proteins. Quantitative analysis of the blots was performed with densitometry scanning. Data from at least three independent experiments were analyzed.

### Reactive Oxygen Species Analysis

The probe CellROX^®^, Green Reagent (Molecular Probe, United States) was applied to detect the cellular reactive oxygen species (ROS) levels. Cells were treated with drug vehicle, Rg3, or 200 μM of tert-butyl hydroperoxide as a positive control for 1 h. Cells were stained with 5 μM probe and 1 μg/ml DAPI for 30 min and then imaged on a laser scanning microscopy (LeicaTC-SP2 Confocal System) using a 40× objective.

### Measurement of Oxygen Consumption Rate

HeLa cells were cultured on Seahorse XF 24 plates for 24 h. To detect oxygen consumption rate (OCR), the growth media was replaced with the XF Assay medium, and the plate was loaded into the Seahorse XF24 Analyzer. OCR baseline measurements were determined for HeLa cells pretreated with Rg3 or vehicle control for 24 h.

### Measurement of ATP Content

Cells were seeded in 96-well plates in triplicate and treated with Rg3 at various doses. After treatment for 24 h, cells were measured with ATPlite kit (Perkin Elmer, United States), according to the instruction (Whitworth et al., 2012). All assays were performed with six replicates in three separate experiments and results are reported as the mean ± SD.

### Mitochondrion Detection

Live cells were incubated with 150 nM Mitotracker Green FM (Life Technologies) for 30 min or with 100 nM Mitotracker Red CMXRos (Life Technologies) for 40 min in the dark at 37°C. Samples were then washed twice in PBS and imaged with a laser scanning microscope (LeicaTC-SP2 Confocal System). The relative fluorescence intensity was quantified by Image J software.

### Statistical Analysis

All experiments were repeated at least three times and representative data were shown. All data were expressed as the mean ± SD. One-way analysis of variance (ANOVA) with multiple comparisons using Dunnett’s test was applied to compare the differences amongst the groups. *P* < 0.05 was considered significantly different.

## Results

### Rg3 Promotes Cell Growth and Proliferation

**FIGURE 1:**
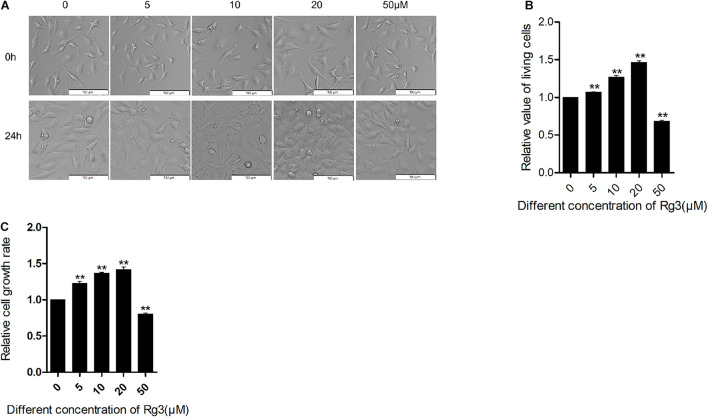
Low concentrations of Rg3 promote cell proliferation. HeLa cells were treated with various concentrations of Rg3 for 24 h in 10% FBS. **(A)** The number and morphology of cells. Cell images were captured using a phase contrast microscope (100×). Scale bar, 100 μm. **(B)** Viable cell count performed using trypan blue at 24 h. **P < 0.01. **(C)** Cell Counting Kit-8 (CCK-8) assay on the effect of Rg3 on cell proliferation. Values shown represent mean ± SD of three independent experiments. One-way ANOVA followed by Dunnett’s *t*-test (**P < 0.01).

Previous studies have demonstrated that Rg3 at concentrations of more than 100 μM inhibits cell growth, but such high concentrations are difficult to achieve *in vivo*. To evaluate how Rg3 affects cell growth at a concentration that is tenable *in vivo*, we treated HeLa cells with different concentrations of Rg3 and examined cell growth and proliferation (Figure 1). We found that while Rg3 inhibited cell proliferation at 50 μM, surprisingly, at concentrations lower than 50 μM, Rg3 stimulated cell proliferation in a concentration -dependent manner (Figure 1B). We further examined the effect of Rg3 on HeLa cell growth using a CCK8 assay (Figure 1C). As expected, in the presence of Rg3 at a concentration lower than 50 μM, the number of cells increased at 24 h. This result demonstrated that Rg3 induces HeLa cell proliferation in a dose-dependent manner.

### Rg3 Activates the mTORC1 and ERK1/2 Signaling Pathways

**FIGURE 2:**
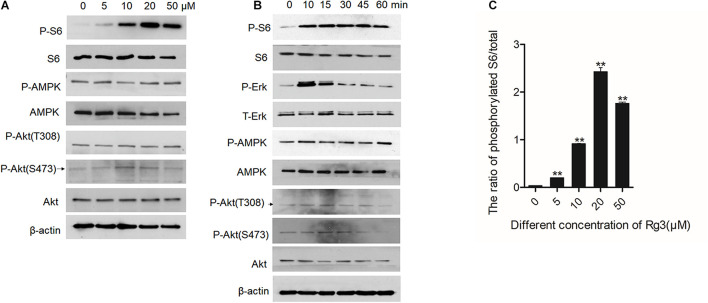
Rg3 activates the mTORC1 and extracellular signal-regulated kinase (ERK)1/2 signaling pathways. Serum starved cells treated with different concentrations of Rg3 for 1 h **(A)** or 20 μM of Rg3 for various time points **(B)**. **(C)** Quantitative presentation of the relative levels of phosphorylated S6 expressed as the ratio of phospho-S6/total S6. Data are from three independent experiments and expressed as mean ± SD. **P < 0.01.

To determine the mechanism by which Rg3 stimulates cell growth, we examined several major growth-promoting signaling pathways in Rg3 treated cells. We found that Rg3 treatment increased mTORC1-dependent phosphorylation of S6 in a dose-dependent manner that peaked at the concentration of 20 μM (Figures 2A,C). However, Rg3 had no obvious effect on activation-dependent phosphorylation of AKT and AMPK (Figure 2A), suggesting that Rg3 activated mTORC1 through an unconventional mechanism. Analysis of the time-dependent activation of mTORC1 revealed that Rg3 was able to activate mTORC1 within 10 min after the cells were exposed to the drug (Figure 2B). We also observed a transient activation of ERK1/2, which peaked at 10 min and then returned to basal level (Figure 2B).

### Rg3 Attenuates Dephosphorylation of S6 Upon Serum Withdrawal

**FIGURE 3:**
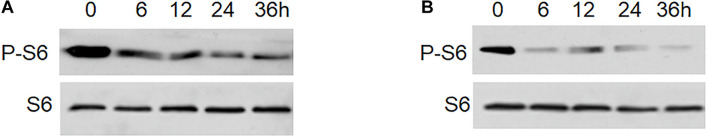
Rg3 attenuates dephosphorylation of S6 upon serum withdrawal. **(A)** Cells were treated with Rg3 **(A)** or vehicle control **(B)** for 30 min followed by serum withdrawal. The levels of phosphorylated S6 and total S6 at indicated time points after the serum withdrawal were determined by Western blotting.

To further determine whether Rg3 stimulates the level of phosphorylated S6 depends on the presence of serum, cells pretreated with or without Rg3 were starved for serum. As showed in the Figure 3B, in cells without Rg3 treatment, the level of phosphorylated S6 decreased rapidly upon serum withdrawal. However, the change of phosphorylated S6 was not obvious in the Rg3 treated cells (Figure 3A).

### Rg3 Stimulates mTORC1 Independent of ROS

**FIGURE 4:**
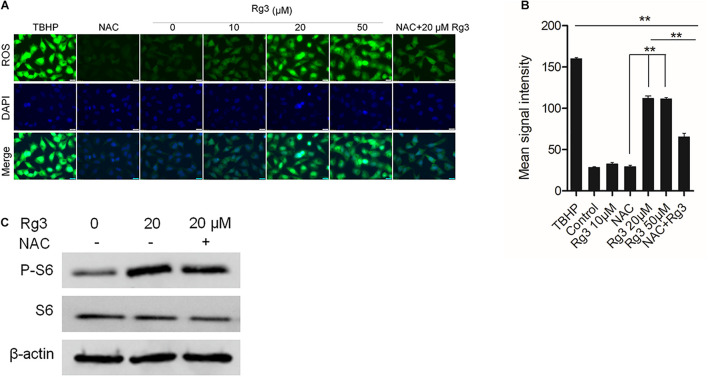
Rg3 stimulates mTORC1 independent of reactive oxygen species (ROS). **(A)** Effect of Rg3 on ROS production. Cells were starved in 0.5% of FBS for 24 h followed by treatment with different concentrations of Rg3 for 1 h. N-acetyl cysteine (NAC) (50 μM) was added to the control and 20 μM Rg3 treated wells. Scale bar, 20 μm. **(B)** Quantitation and statistical analysis of oxidative stress based on staining with CellROX^®^, Oxidative Stress Reagents. Data are from three independent experiments and expressed as mean ± SD. **P < 0.01. **(C)** Cells were pretreated with 50 μM NAC (+) or vehicle control (–) for 10 min followed by treatment with Rg3 for 1 h. mTORC1-dependent phosphorylation of S6 was determined by Western blot.

The above results indicated that Rg3 affects mTORC1 independent of AKT and AMPK, We further examined if ROS, which stimulates mTORC1 activity at low concentrations (Martin et al., 2013), mediates the effect of Rg3 on the activation of mTORC1. We first evaluated whether Rg3 stimulates ROS production. We found that in comparison with vehicle-treated cells, cells treated with Rg3 at a concentration higher than 10 μM exhibited a strong accumulation of ROS (Figures 4A,B). Rg3 induced increase in ROS was blocked by treatment with the reducing agent, NAC, However, NAC had no significant effect on the P-S6 level stimulated by Rg3 (Figure 4C), suggesting that the activation of mTORC1 by Rg3 was independent of ROS.

### Rg3 Promotes Mitochondrial Biogenesis and Function

**FIGURE 5:**
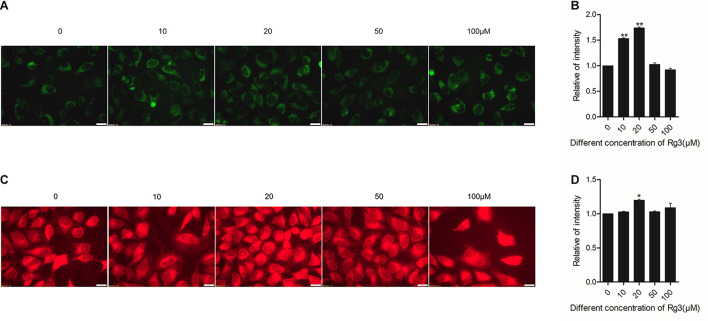
Rg3 promotes mitochondrial biogenesis and function. Cells were treated with different concentrations of Rg3 for 24 h. **(A)** Cells were stained with 150 nM Mitotracker Green FM for 30 min and imaged with fluorescent microscopy. Scale bar, 100 μm. **(B)** Quantitative presentation of the fluorescent intensity on mitochondria shown in panel **(A)**. Data were collected from three independent experiments and expressed as mean ± SD. **P < 0.01 **(C)** Cells were stained with MitoTracker Red CMXRos (100 nM) for 40 min and imaged with fluorescent microscopy. Scale bar, 100 μm. **(D)** Quantitative presentation of the fluorescent intensity of mitochondria shown in panel **(C)**. Data were collected from three independent experiments and expressed in mean ± SD. *P < 0.05.

Next, we examined the effect of Rg3 on the biogenesis of mitochondria and their function. We used a membrane potential-(Δψm)-independent mitochondrial stains, MitoTracker Green FM, to measure the number of mitochondria and Δψm-dependent dye MitoTracker Red CMXRos to monitor mitochondrial integrity. We found that Rg3 at concentrations lower than 50 μM dose-dependently increased the number of mitochondria. However, the drug had an opposite effect at higher concentrations (Figures 5A,B). Rg3 at 20 μM also caused a small but significant increase in mitochondrial membrance potential- (Figures 5C,D), suggesting that the drug was able to affect mitochondrial activity. Collectively, these findings demonstrated that Rg3 can increase the number of mitochondria and affect the activity of mitochondria.

### Rg3 Increases the Basic Oxygen Consumption and ATP Generation

**FIGURE 6:**
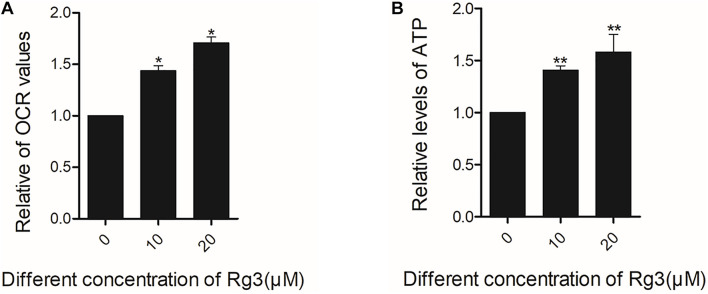
Rg3 increases the basic oxygen consumption and ATP of cells. Cells were treated with Rg3 for 24 h with 10% FBS. **(A)** The basal oxygen consumption rate (OCR) value were obtained using a Seahorse Extracellular Flux Analyzer. *P < 0.05. **(B)** the levels of ATP in the treated cells was measured. The data were from three independent experiments and expressed in mean ± SD. The differences between the control and Rg3 treated samples. **P < 0.01.

Oxygen consumption rate is a measure of the cellular respiration and mitochondrial function. Using a Seahorse Extracellular Flux Analyzer, we monitored the cellular OCR of cells treated with Rg3 in real time. We found that Rg3 treatment significantly increased the OCR of the cells (Figure 6A). Similarly, Rg3 also increased the levels of ATP in the treated cells (Figure 6B). These results indicated that Rg3 can increase the basic oxygen consumption and ATP production.

## Discussion

As a ginsenosides monomer, Rg3 has been reported to be beneficial for the treatment of various cancers t. Previous studies indicate that Rg3 has potential as a chemo preventive agent or adjuvant treatment. *In vitro* studies have shown that Rg3 at high concentrations (>100 μM) is able to inhibit cancer cell proliferation and migration (Zhao et al., 2019). However, Rg3 has poor oral bioavailability mainly because of its extensive pre-systemic metabolism and poor membrane permeability (Jia et al., 2009). Thus, it is very difficult to achieve the concentration of Rg3 *in vivo* required for inhibiting cancer cell growth. Therefore, further *in vivo* studies are required to elucidate the beneficial effects of Rg3 in the treatment of tumors.

In the present study, we show that Rg3 at low concentrations is able to stimulate, instead of inhibiting, cell growth. We find that Rg3 has an immediate effect on activating the mTORC1 and MAPK signaling pathways. Surprisingly, Rg3 does not affect the activities of AKT and AMPK, two upstream regulators of mTORC1 that, respectively, channel growth factor and energy signals to mTORC1. This observation suggests that Rg3 activates mTORC1 independent of the conventional mechanisms. We find that Rg3 can stimulate cellular ROS production. Although it is possible that Rg3 activates mTORC1 through increasing ROS levels by inducing, oxidative stress, we consider this possibility unlikely for the following two reasons. First, Rg3 could activate mTORC1 at low concentrations that had no obvious effects on cellular ROS levels (Figure 2B). Second, pretreating cells with NAC prevented Rg3-induced ROS accumulation but failed to block mTORC1 activation. These observations indicate that Rg3 activates mTORC1 through a novel mechanism. The immediate effect of Rg3 on mTORC1 activation also indicates that Rg3 may act on mTORC1 through a direct mechanism.

At the concentrations lower than 50 μM, Rg3 also drastically increases the number of mitochondria (Figure 5B), which is accompanied by an enhancement in oxygen consumption and the intracellular ATP level (Figure 6B). The increase in the number of mitochondria is likely to be a consequence of the Rg3-stimulated mTORC1 activation, which has been previously shown to promote mitochondrial biogenesis (Larsson et al., 1985). The high level of mitochondria is expected to lead to an elevated oxygen consumption and ATP production.

The elevated activity of mTORC1 in Rg3 treated cells is consistent with the higher rate of cell proliferation. To bolster cellular proliferation and growth, mTORC1 stimulates biosynthetic processes including protein synthesis, and acts as a primary regulator of energy production in mitochondria. The synthesis of proteins is positively correlated with the cell proliferative rate (Larsson et al., 1985). In turn, mitochondrial ATP production is required to fuel protein synthesis and proliferation (Buttgereit and Brand, 1995; Rolfe and Brown, 1997). The correlation between the elevated mTORC1 activity and increased cell proliferation in Rg3 treated cells indicates that Rg3 may act through enhancing mTORC1 signaling activities to promote cell growth and survival.

## Conclusion

In conclusion, our data suggest that Rg3 at low concentrations is able to promote cell growth through activation of mTORC1. This effect is in contrast with that of high concentrations of Rg3, which causes cell death. Given the low bioavailability of Rg3, it is expected to be difficult to achieve high concentrations of Rg3 *in vivo* that could exhibit the same cytotoxic effects *in vitro*. In this regard, the benefit effects of Rg3 in cancer treatment may lie on its activity in promoting the recovery of normal cells, such as gastric stem cells and hematopoietic precursor cells after chemotherapies. Therefore, the use of Rg3 compound as an anticancer agent should be evaluated with caution.

## Data Availability Statement

The original contributions presented in the study are included in the article/Supplementary Material, further inquiries can be directed to the corresponding authors.

## Author Contributions

YJ and L-ZL designed this study. WL, S-XZ, DZ, L-HH, and BA carried out most of the experiments and wrote the manuscript. Z-SC, L-LS, and H-FP supervised the experiments, analyzed the results, and proofread the manuscript. All authors have read and agreed with the submitted version of the manuscript.

## Conflict of Interest

The authors declare that the research was conducted in the absence of any commercial or financial relationships that could be construed as a potential conflict of interest.

## Publisher’s Note

All claims expressed in this article are solely those of the authors and do not necessarily represent those of their affiliated organizations, or those of the publisher, the editors and the reviewers. Any product that may be evaluated in this article, or claim that may be made by its manufacturer, is not guaranteed or endorsed by the publisher.
