# Supplementary material for: Ginsenoside Rg3 Promotes Cell Growth Through Activation of mTORC1
Source: Front Cell Dev Biol. 2021 Sep 13;9:730309. doi: 10.3389/fcell.2021.730309 (PMC8473834; doi:10.3389/fcell.2021.730309)
Supplement: Supplementary file 1 [file Data_Sheet_1.PDF]

Supplementary Material

Supplementary Figures

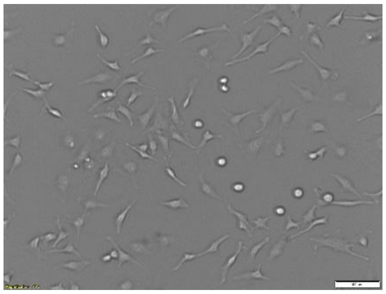<br>**0 µ M of Rg3(0h)**	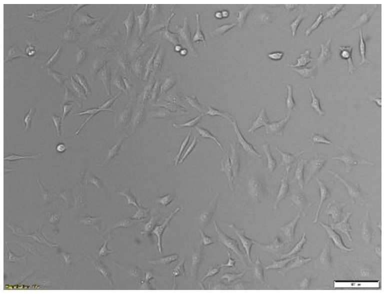<br>**0 µ M of Rg3(0h)**	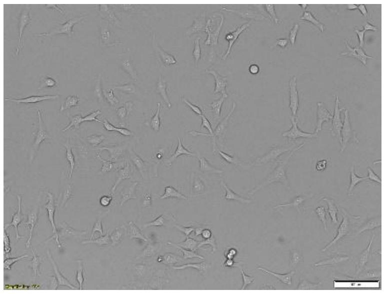<br>**0 µ M of Rg3(0h)**
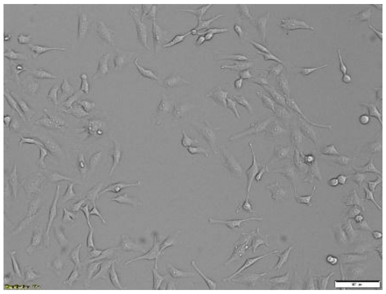<br>**0 µM of Rg3(0h)**	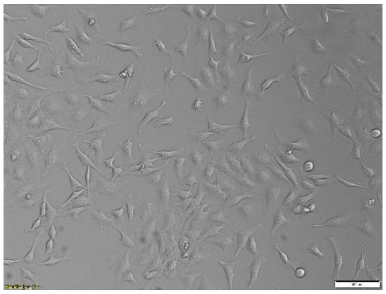<br>**0 µM of Rg3(0h)**	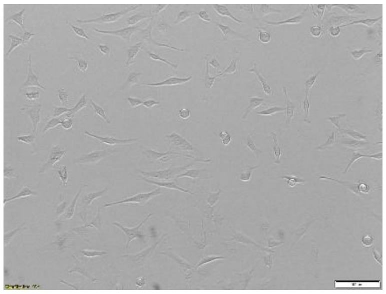<br>**0 µM of Rg3(0h)**
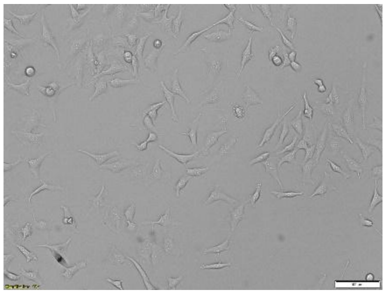<br>**0 µM of Rg3(0h)**	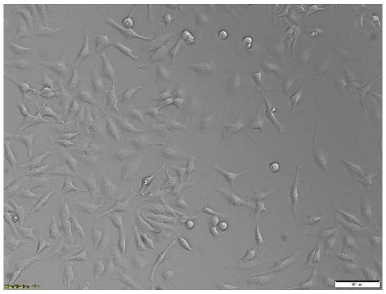<br>**0µM of Rg3(0h)**	

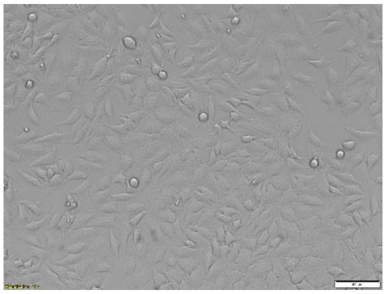 **0 µM of Rg3 (24h)**	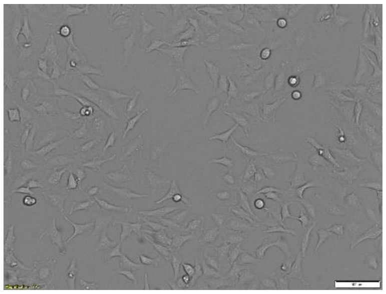 **0 µM of Rg3 (24h)**	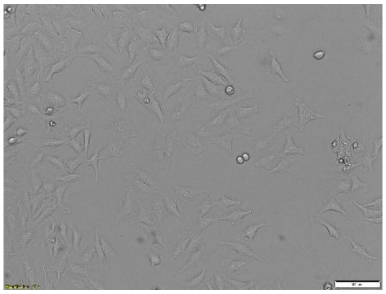 **0 µM of Rg3 (24h)**
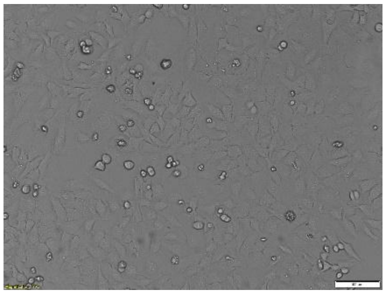 **5 µM of Rg3(24h)**	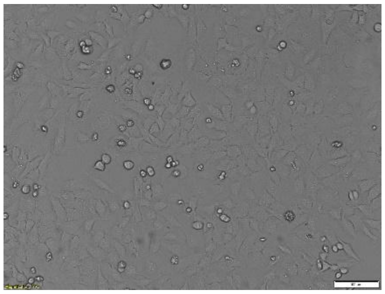 **5 µM of Rg3(24h)**	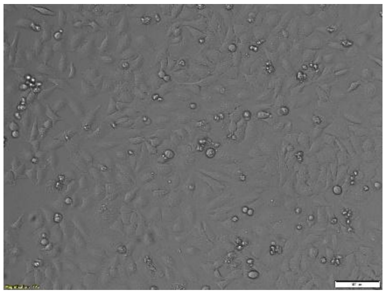 **5 µM of Rg3(24h)**

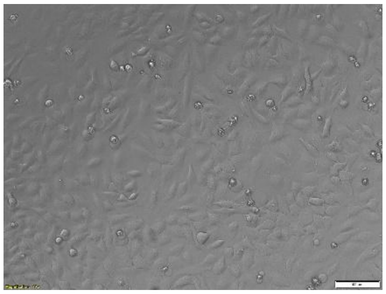<br>**10 µM of Rg3(24h)**	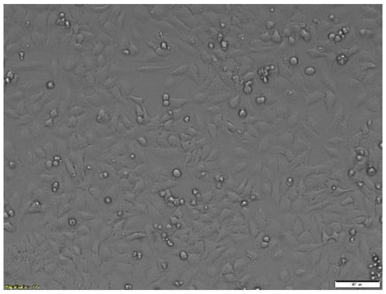<br>**10 µM of Rg3(24h)**	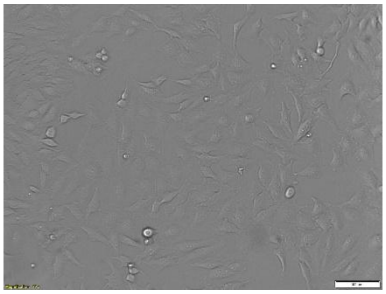<br>**10 µM of Rg3(24h)**
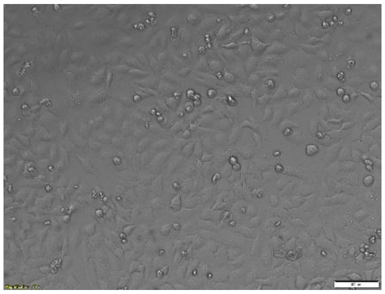<br>**20µM of Rg3(24h)**	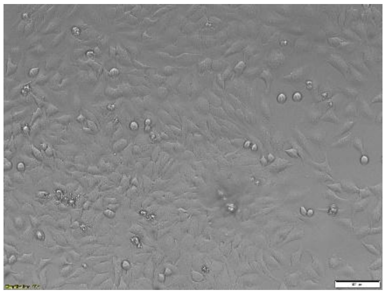<br>**20µM of Rg3(24h)**	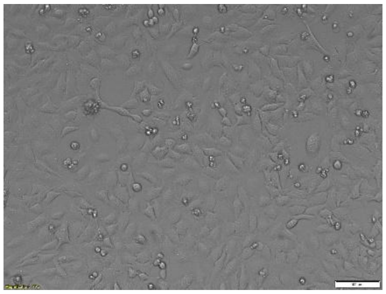<br>**20µM of Rg3(24h)**
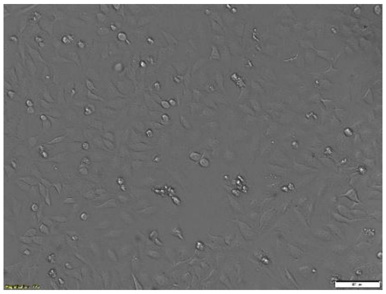<br>**50µM of Rg3(24h)**	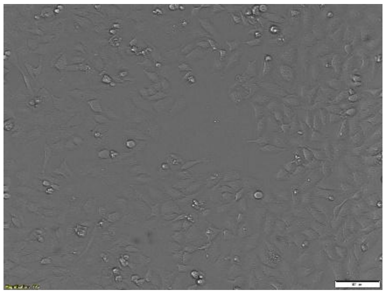<br>**50µM of Rg3(24h)**	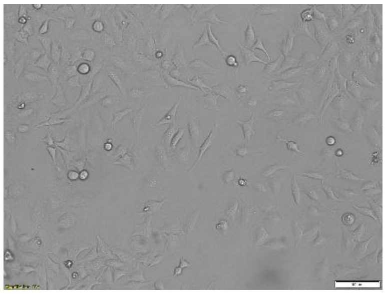<br>**50µM of Rg3(24h)**

Supplementary Figure 1A. For the results of each picture, we repeat the experiment at least three times, and three of the results are selected as supplementary pictures here.

Relative value of living cells

group			
0µM	1	1	1
5µM	1.06	1.08	1.07
10µM	1.13	1.23	1.3
20µM	1.33	1.5	1.46
50µM	0.66	0.68	0.71

Supplementary Figure 1B.

Relative cell growth rate

OD\Concentration of Rg3	0	5	10	20	50	Blank
	1.44	1.588	1.817	1.862	1.254	0.183
	1.412	1.751	1.881	1.738	1.115	0.186
	1.405	1.773	1.858	1.988	1.121	0.185
	1.406	1.675	1.866	1.939	1.23	0.188
	1.406	1.687	1.879	1.965	1.161	0.183
	1.396	1.666	1.856	2.019	1.127	0.184

Supplementary Figure 1C.

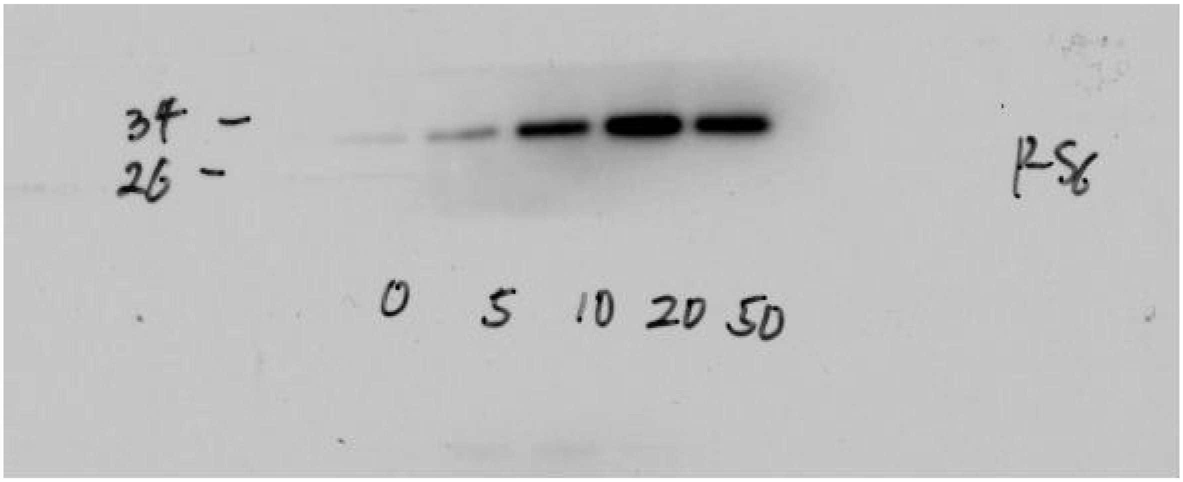

Supplementary Figure 2A. P-S6

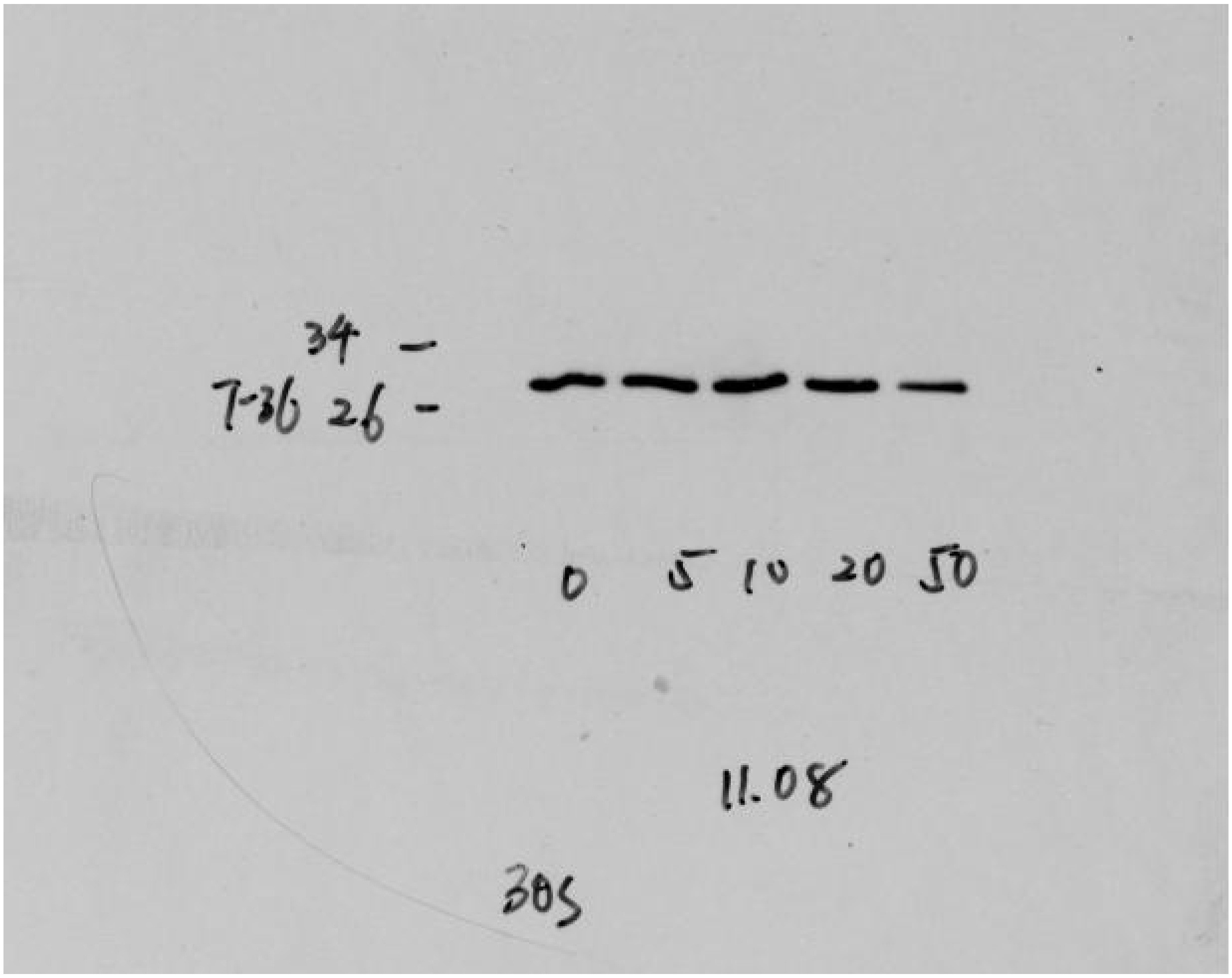

Supplementary Figure 2A. S6

IntDen	0μM Rg3	5μM Rg3	10μM Rg3	20μM Rg3	50μM Rg3
PS6	483.799	2600.518	12741.681	30693.9467	20907.095
S6	12976.51	12995.832	13885.317	11811.447	12035.539
PS6/S6 Relative intensity	0.037313957	0.199551222	0.911363236	2.598661	1.737113311

Supplementary Figure 2C.

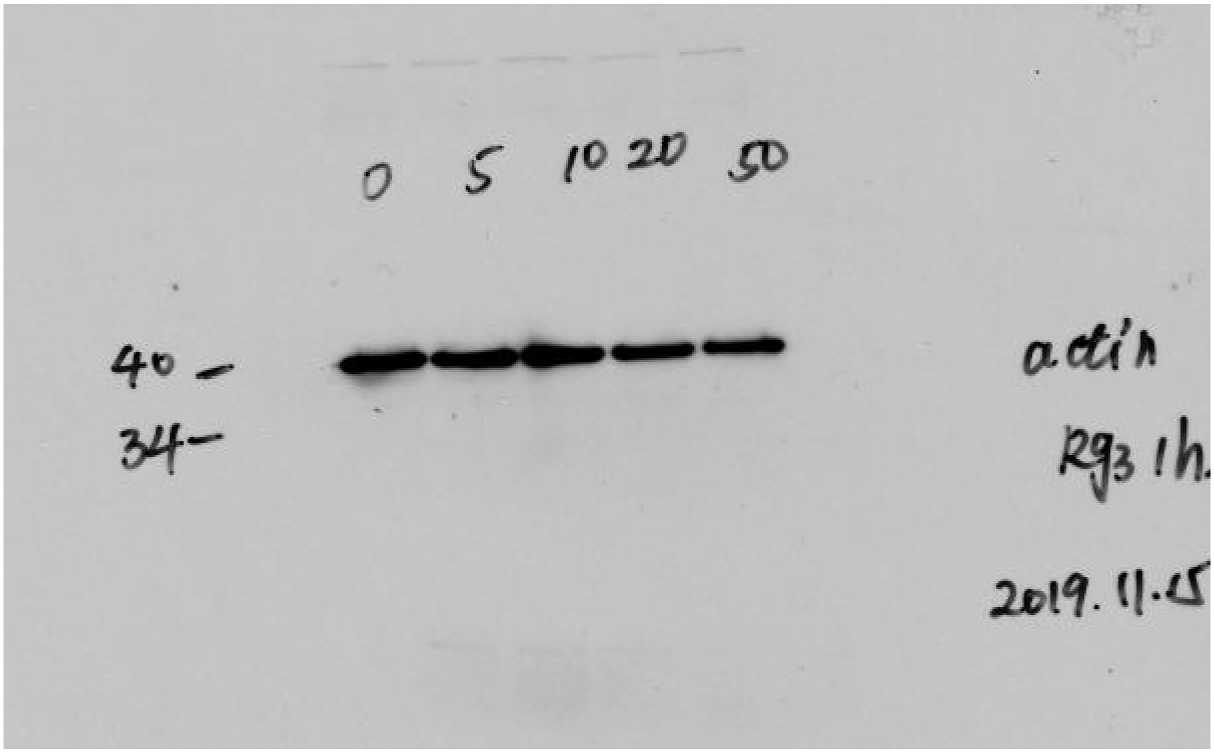

Supplementary Figure2A. β actin

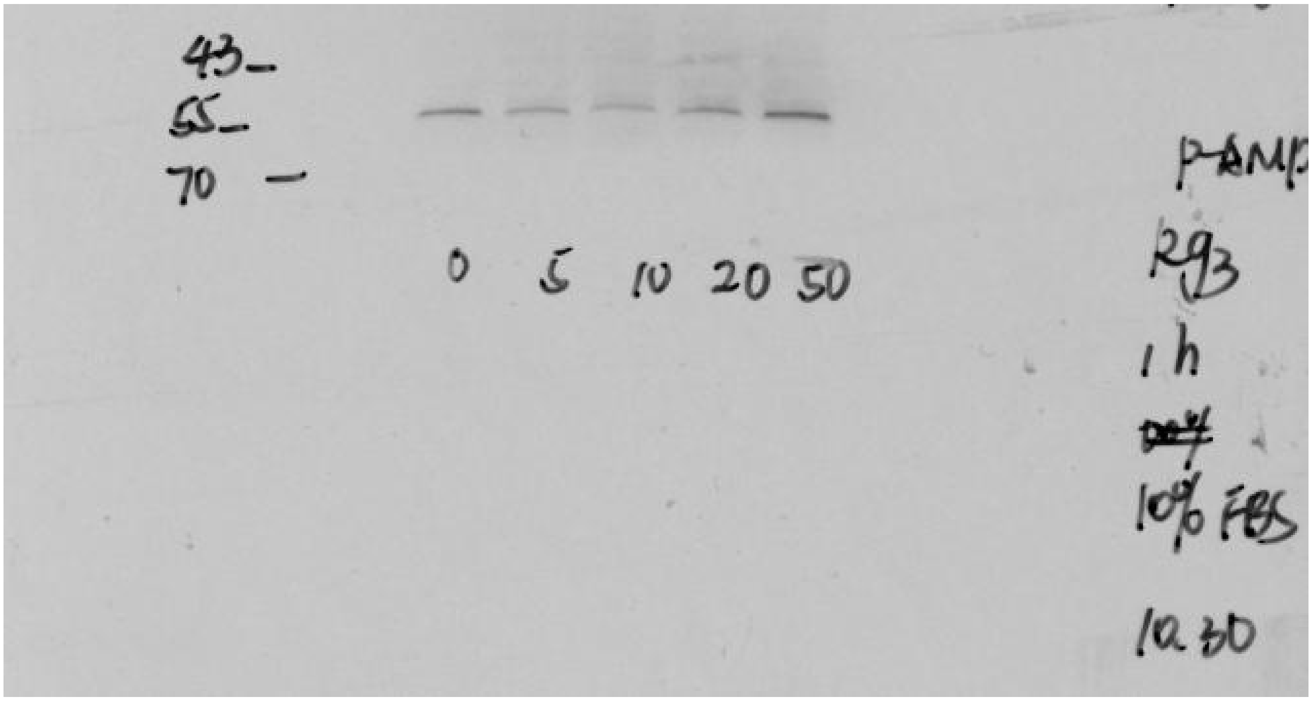

Supplementary Figure2A. P-AMPK

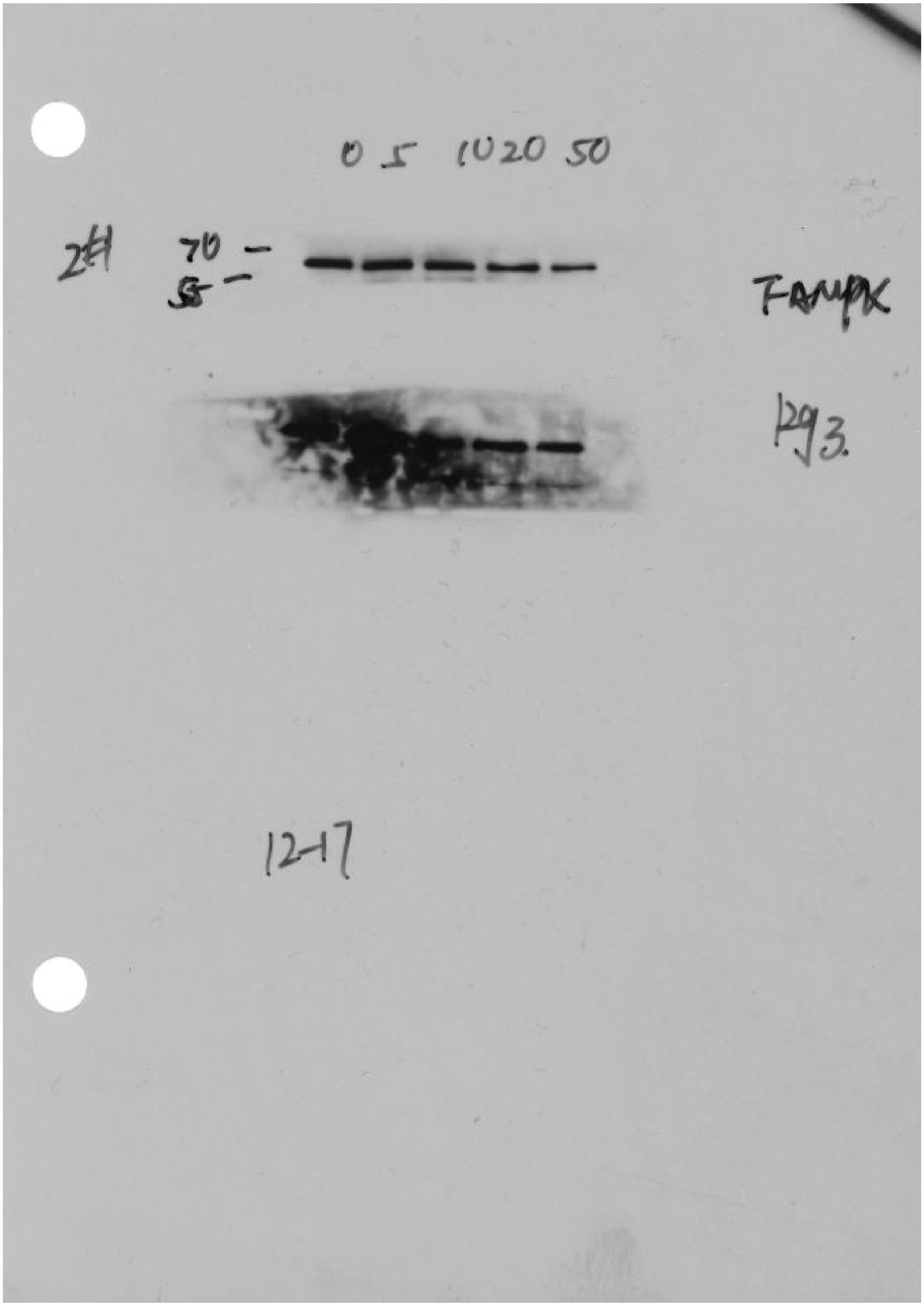

Supplementary Figure2A. T-AMPK

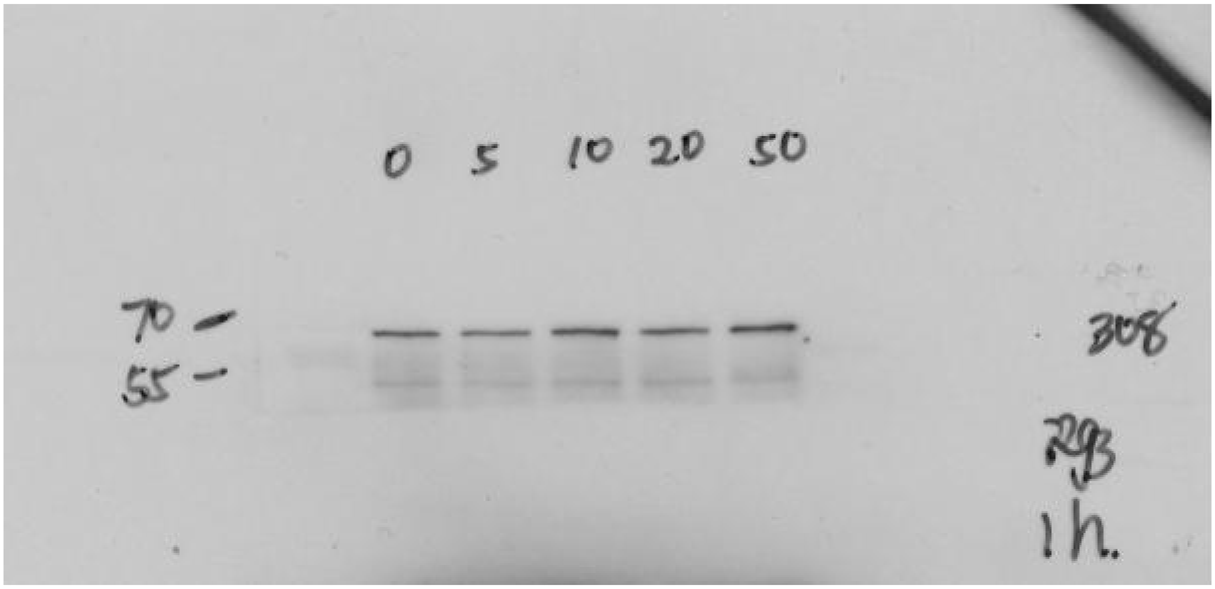

Supplementary Figure 2A. P-AKT 308

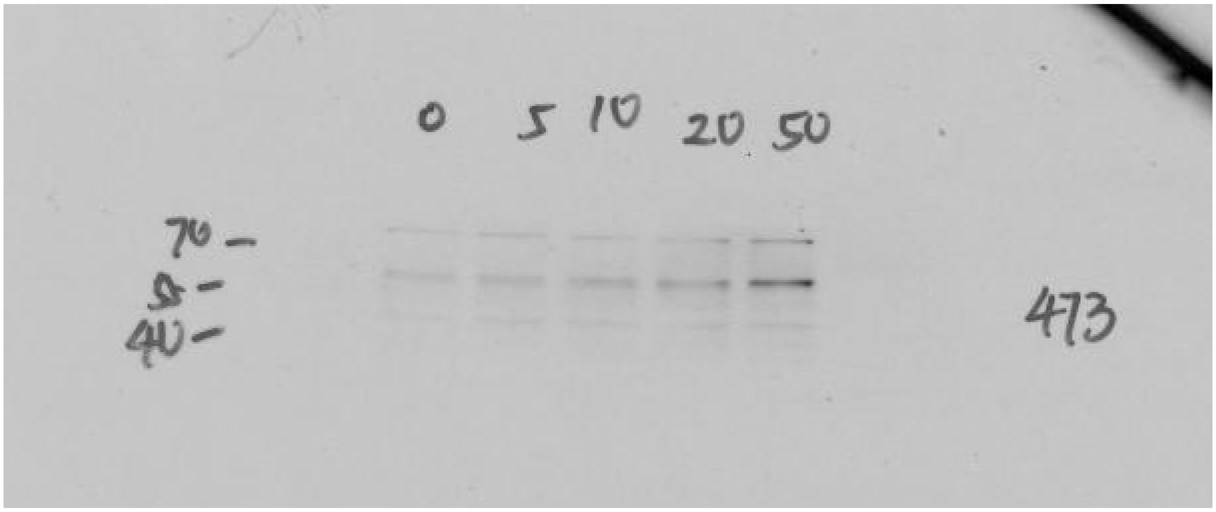

Supplementary Figure 2A. P-AKT 473

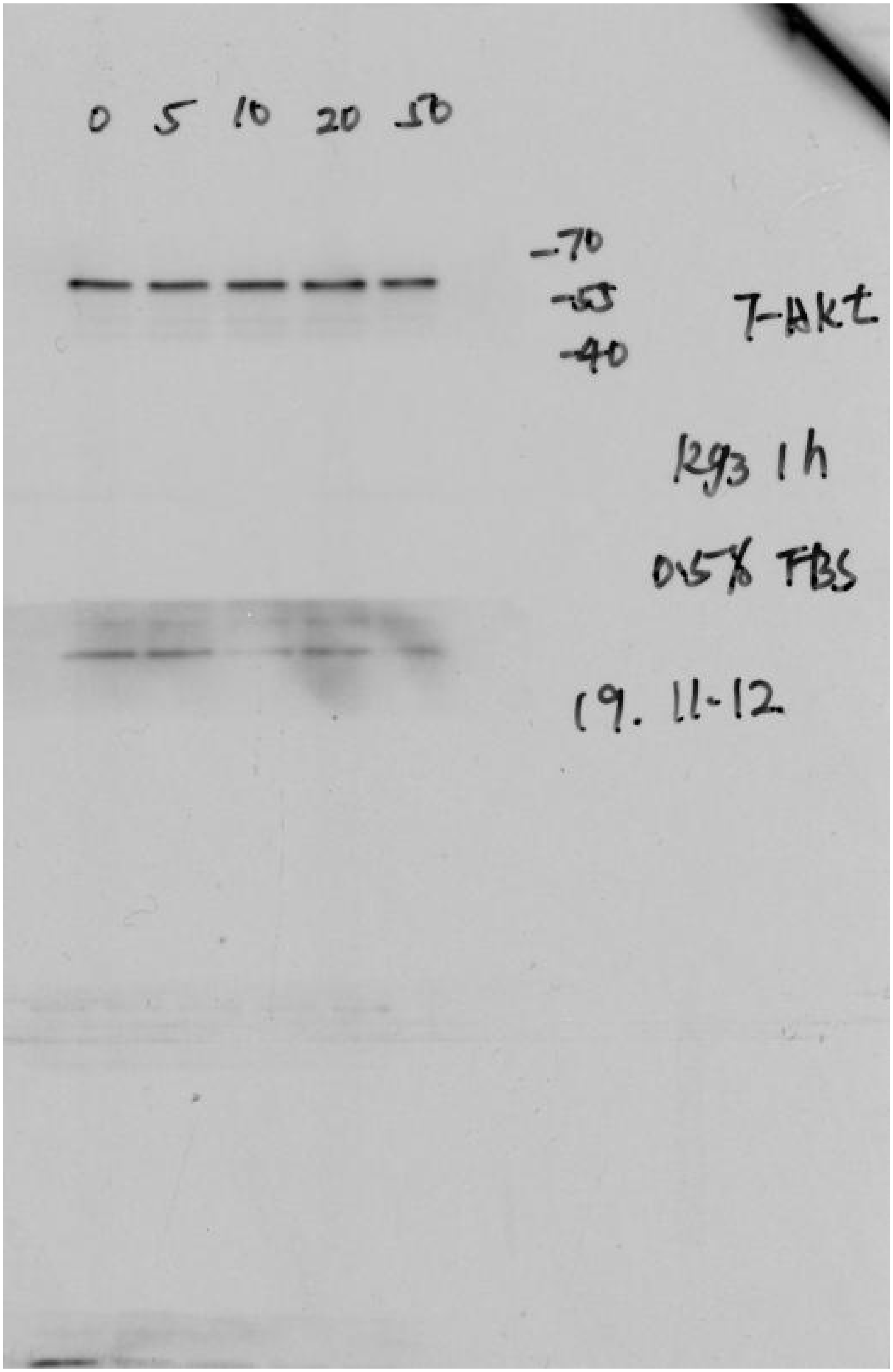

Supplementary Figure 2A. T-AKT

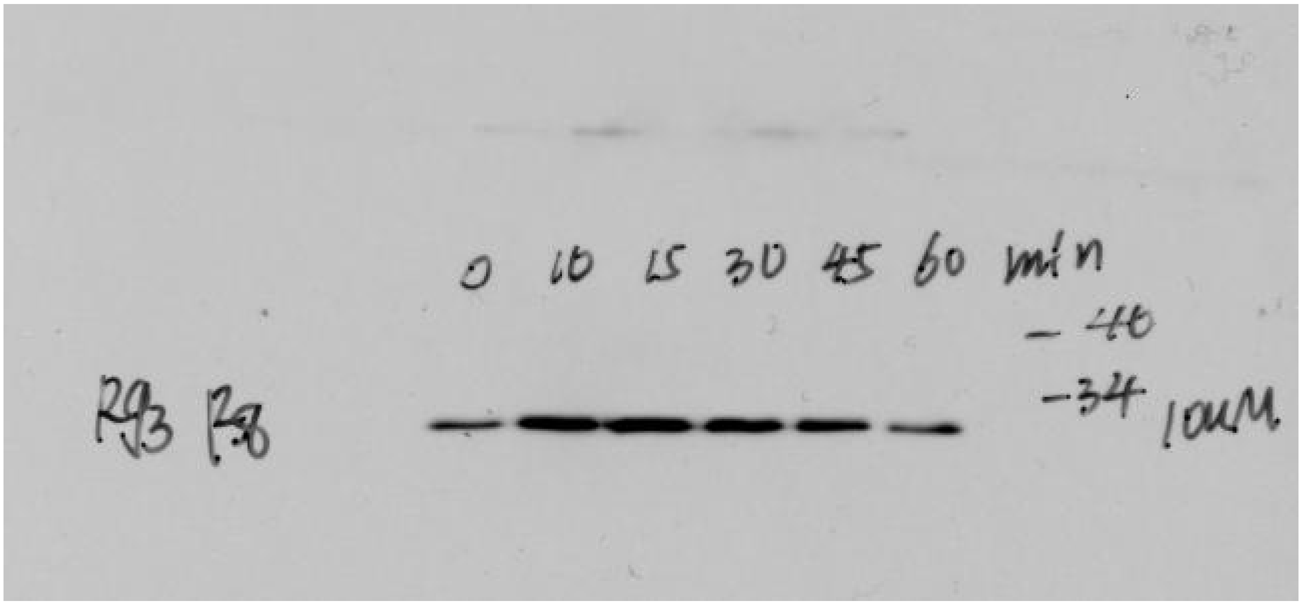

Supplementary Figure 2B. P S6

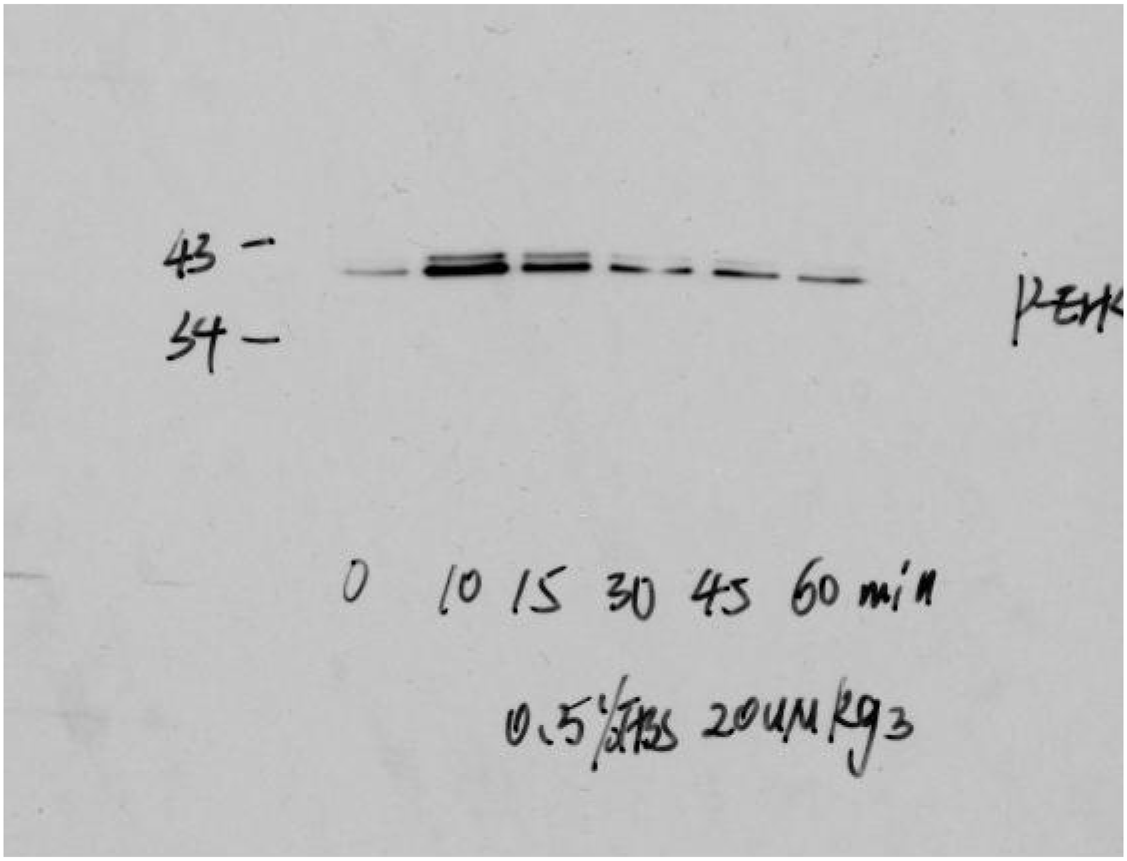

Supplementary Figure 2B. P Erk

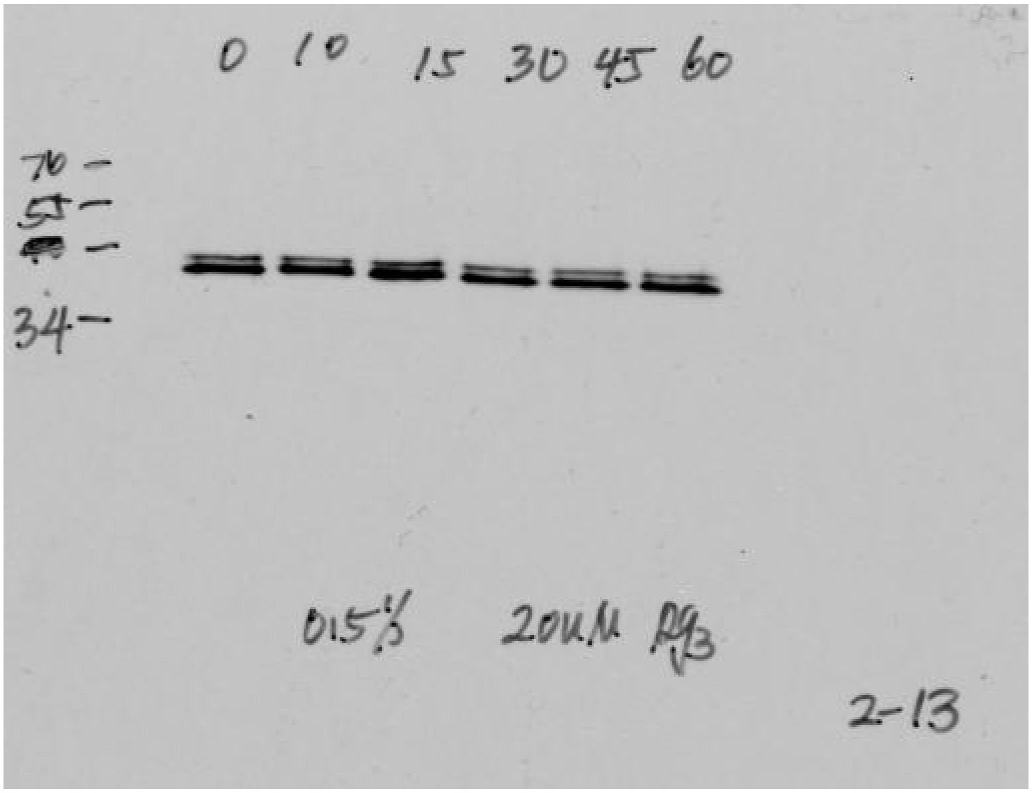

Supplementary Figure 2B. T-Erk

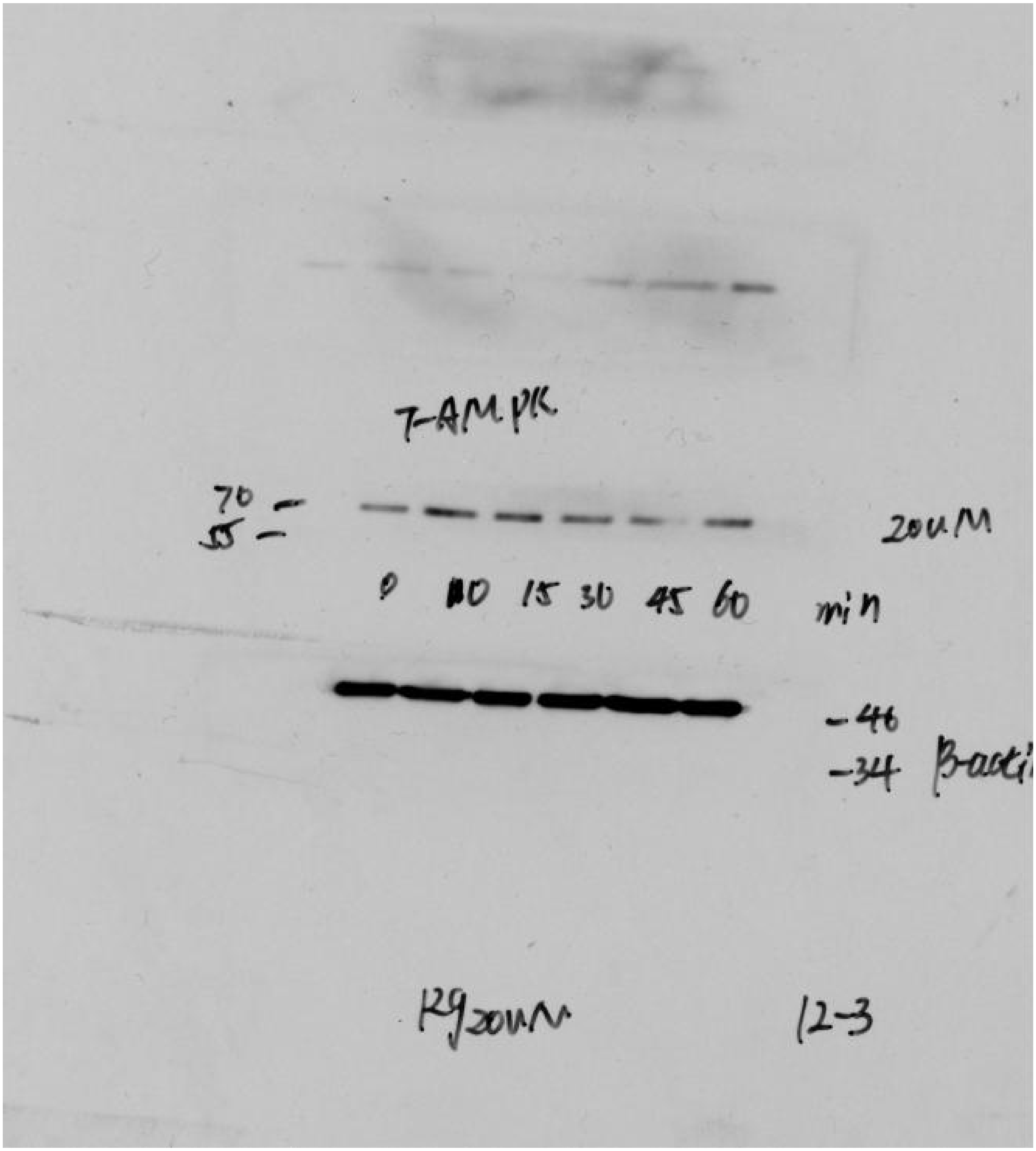

Supplementary Figure 2B. β actin

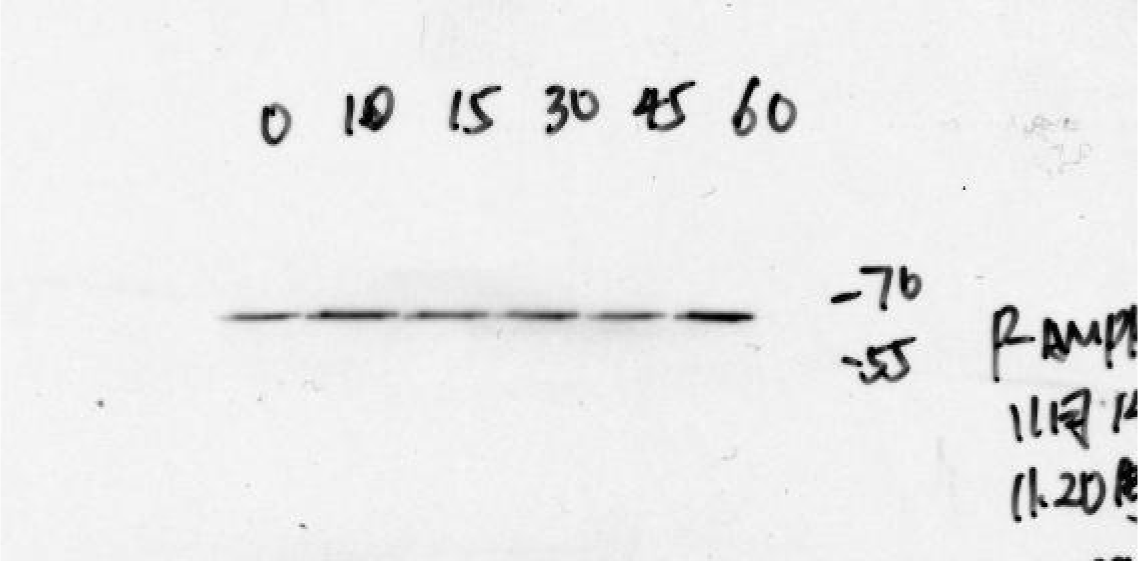

Supplementary Figure 2B. P AMPK

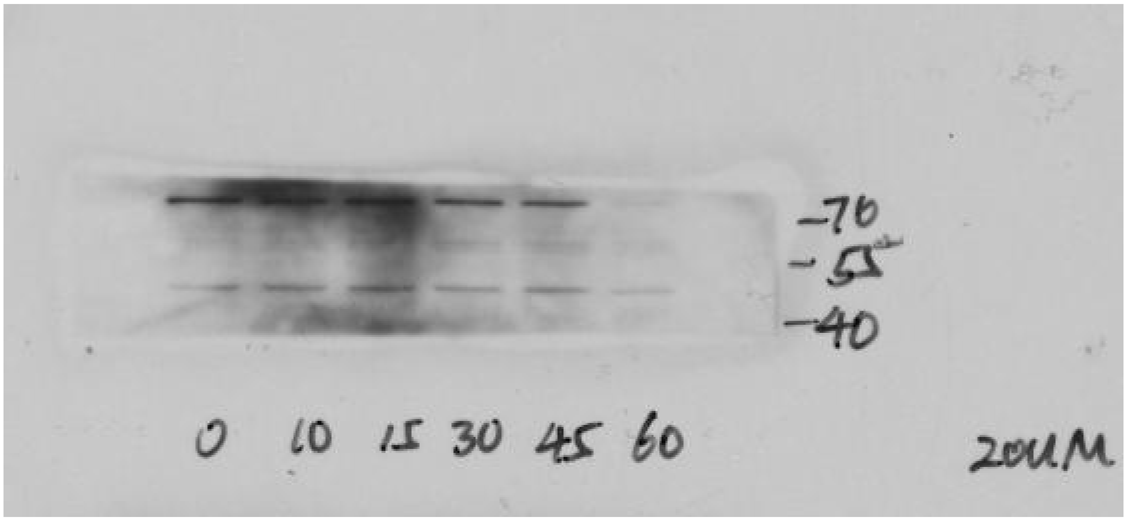

Supplementary Figure 2B. P-Akt308

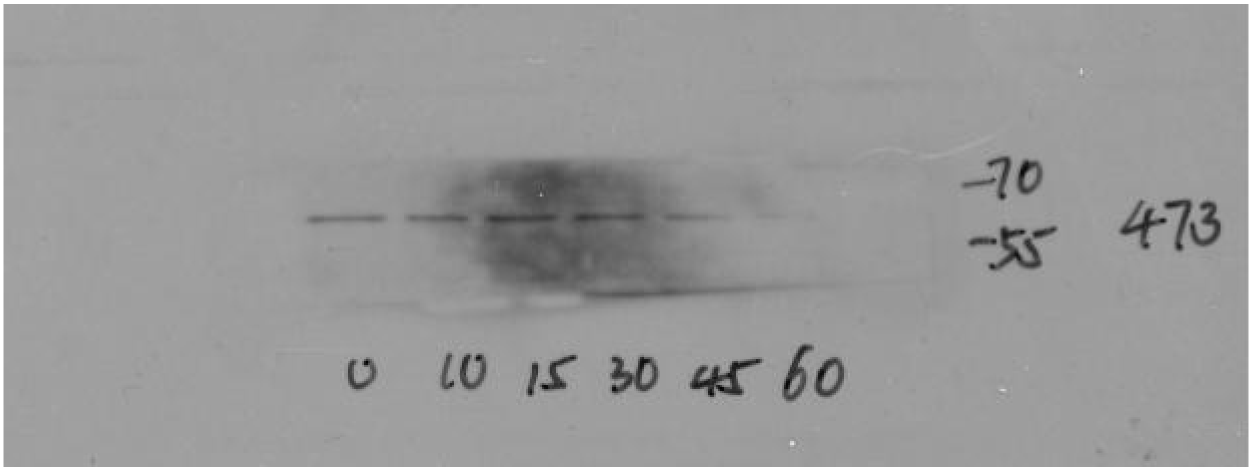

Supplementary Figure 2B. P-Akt473

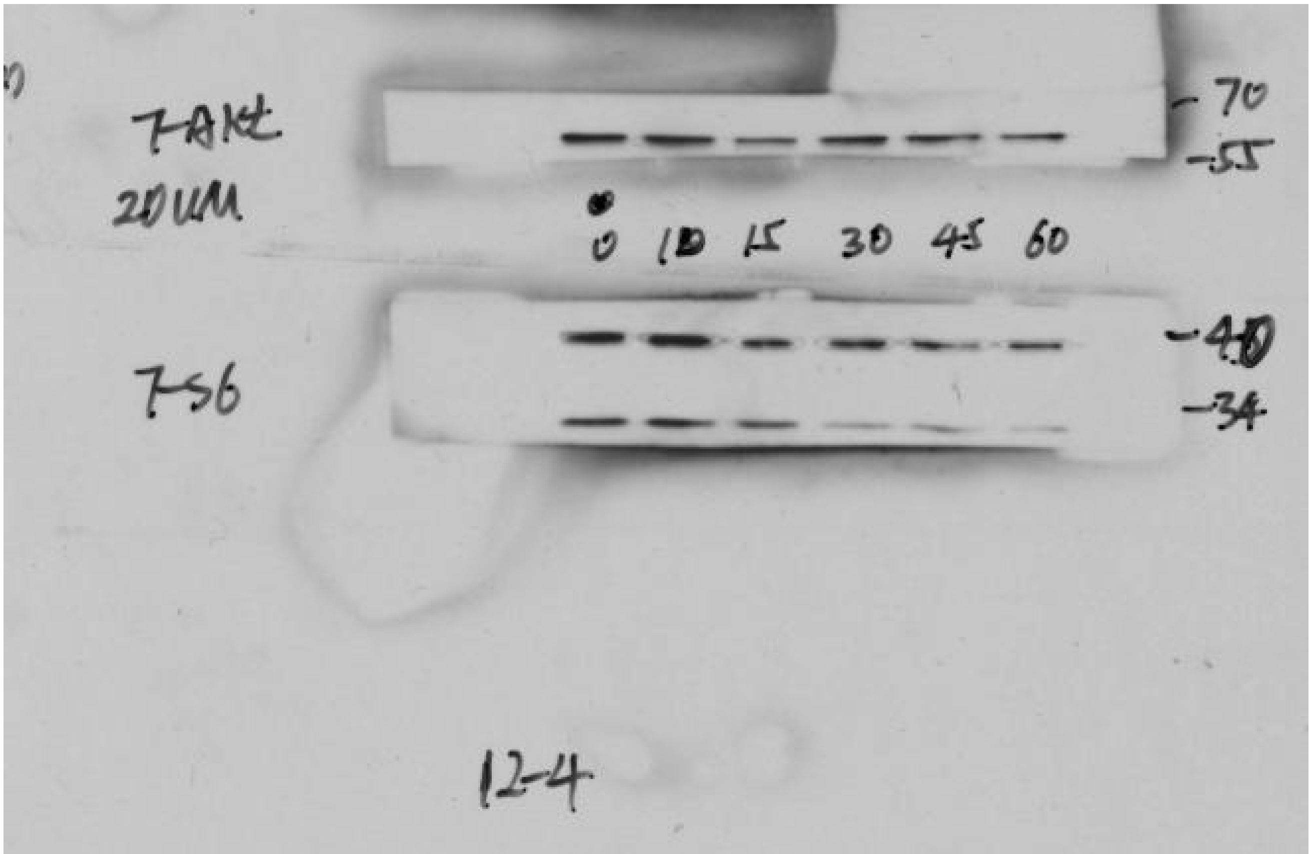

Supplementary Figure 2B. T-Akt and S6

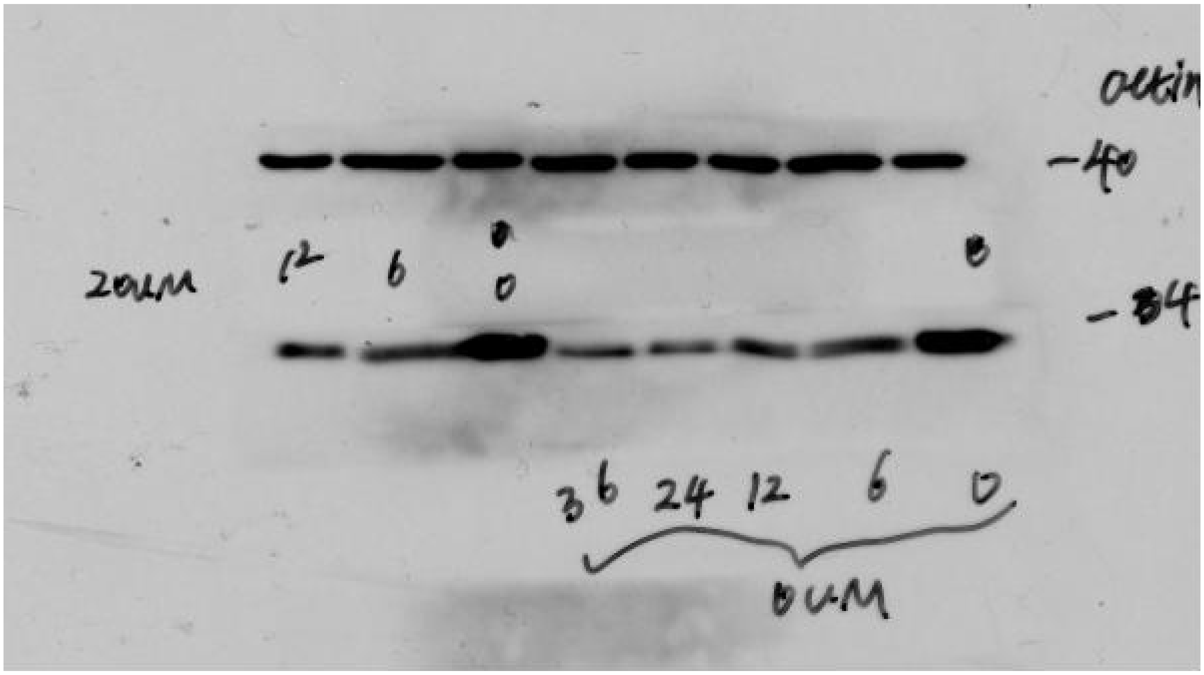

Supplementary Figure 3A.β actin P-S6

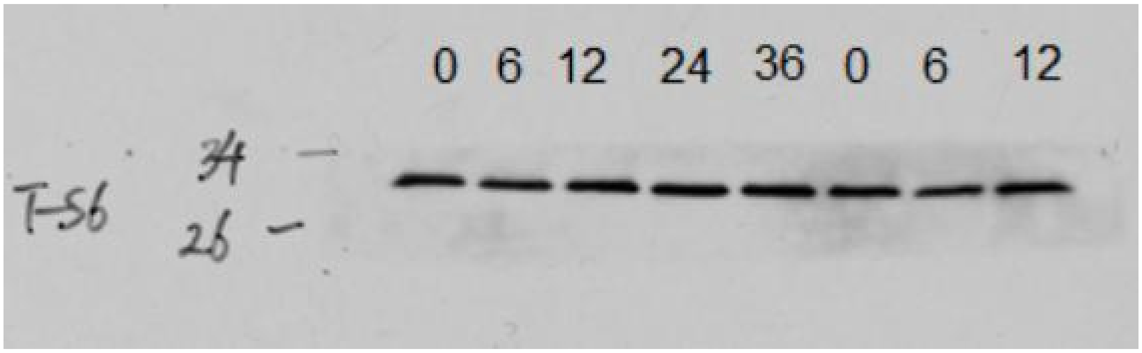

Supplementary Figure 3A.S6

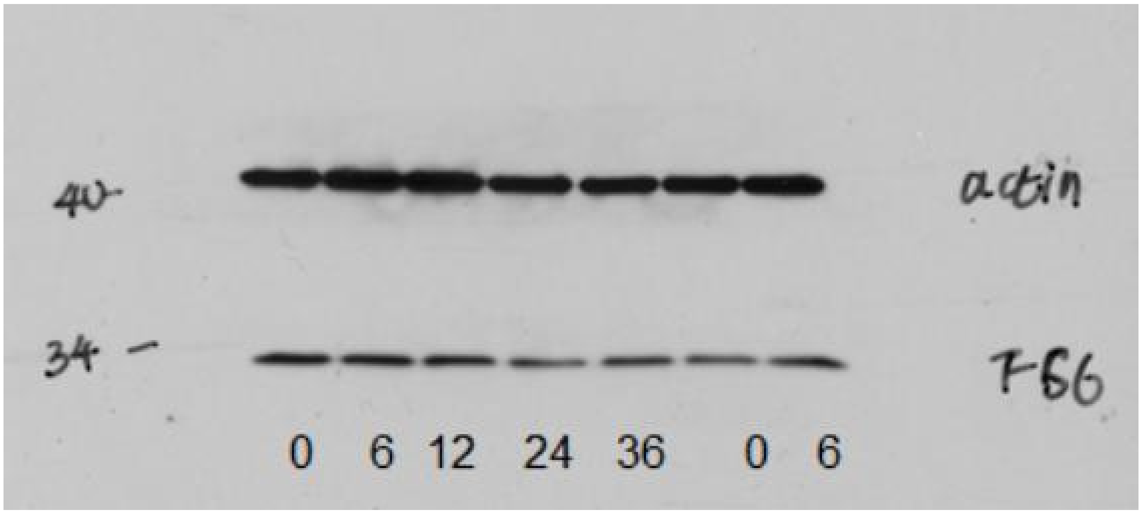

Supplementary Figure 3B.β actin and S6

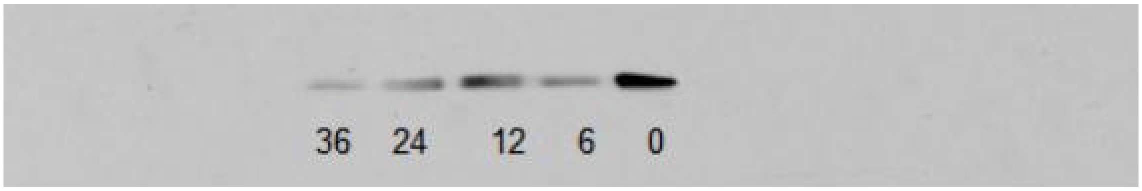

Supplementary Figure 3B. P-S6

**ROS**	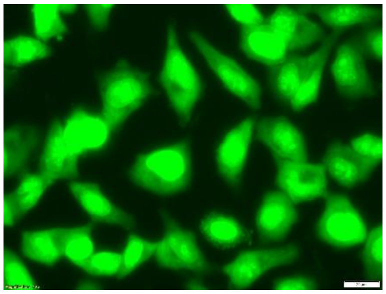	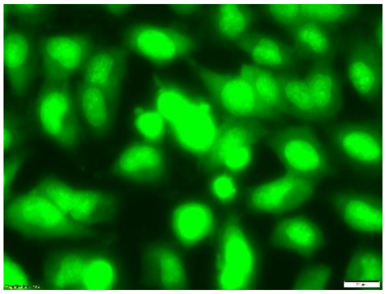
**DAPI**	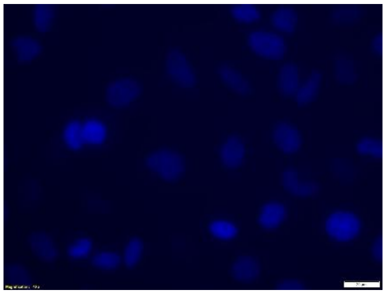	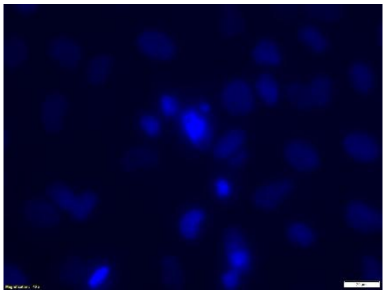
**Merge**	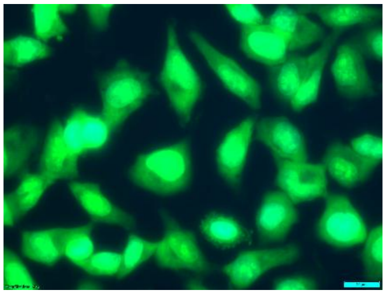	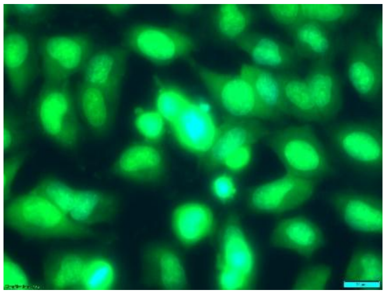

Supplementary Figure 4A. TBHP Each experiment has been repeated twice

**ROS**	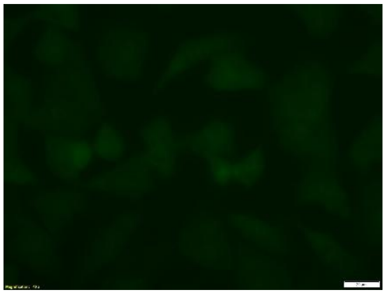	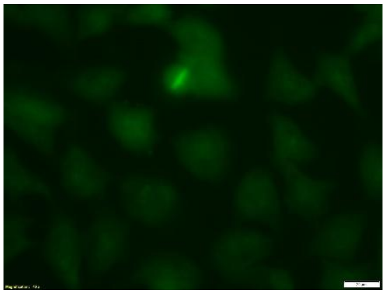
**DAPI**	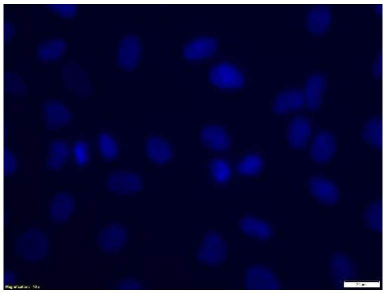	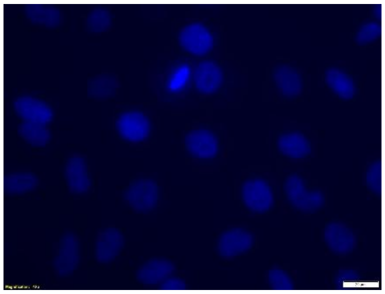
**Merge**	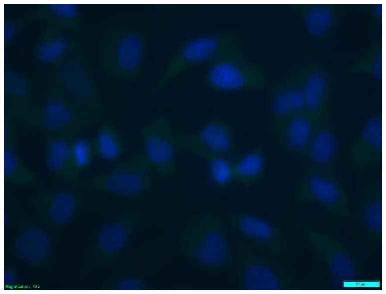	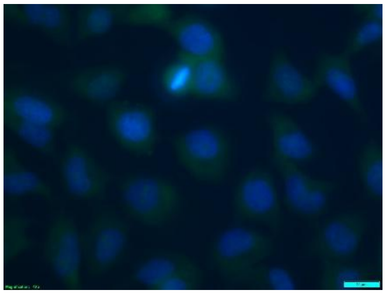

Supplementary Figure 4A. NAC Each experiment has been repeated twice

**ROS**	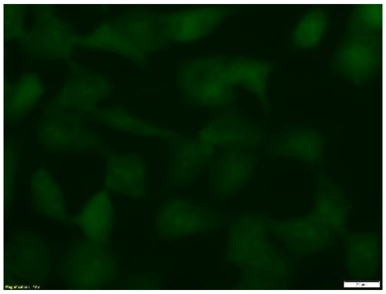	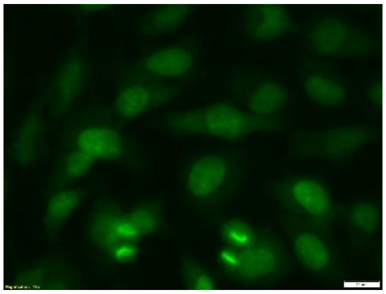
**DAPI**	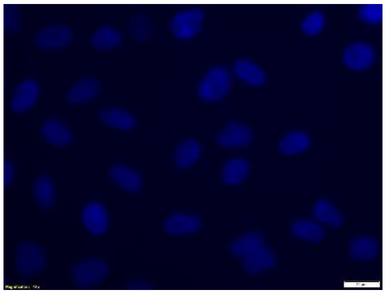	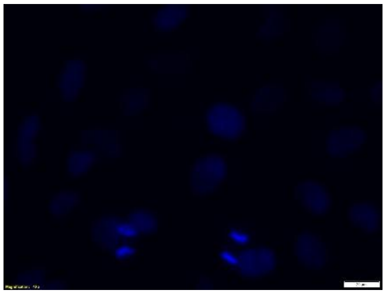
**Merge**	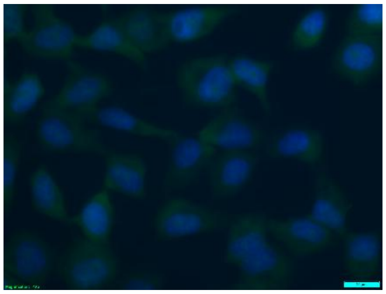	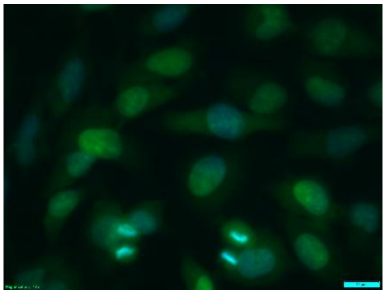

Supplementary Figure 4A. 0 µM Rg3 Each experiment has been repeated twice

**ROS**	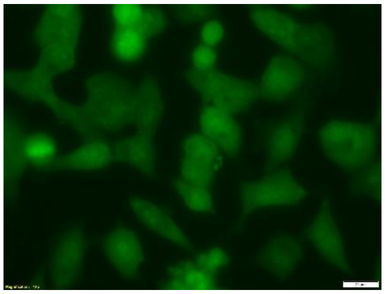	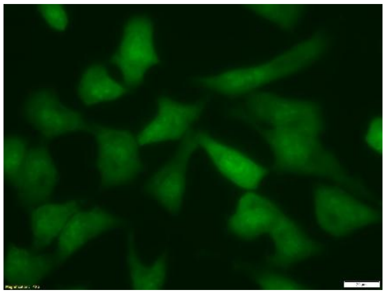
**DAPI**	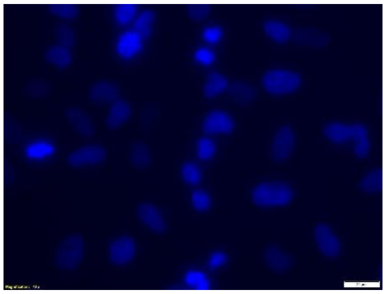	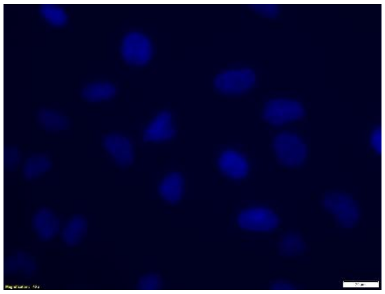
**Merge**	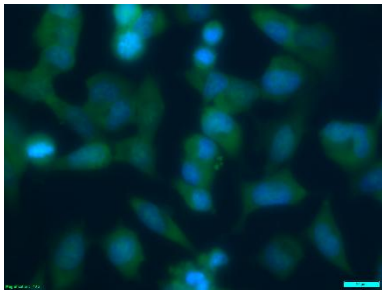	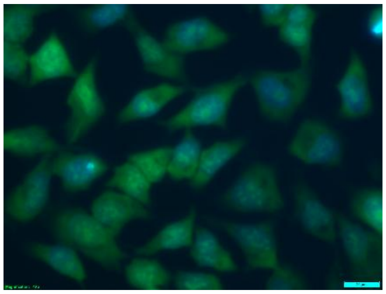

Supplementary Figure 4A. 10 µM Rg3   Each experiment has been repeated twice

**ROS**	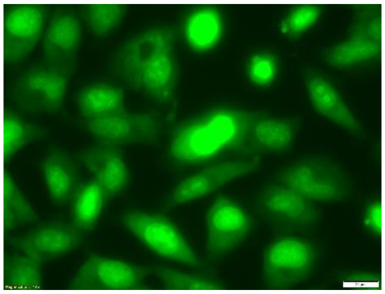	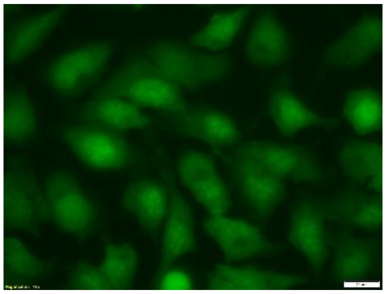
**DAPI**	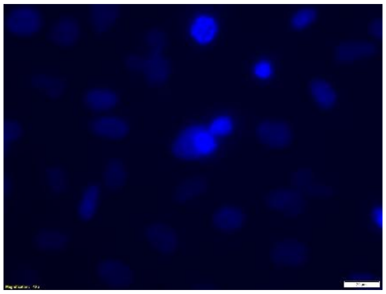	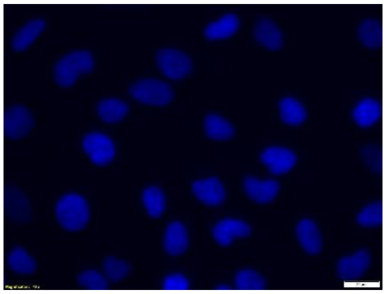
**Merge**	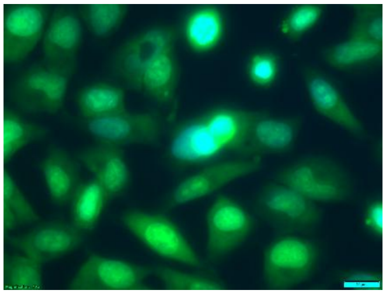	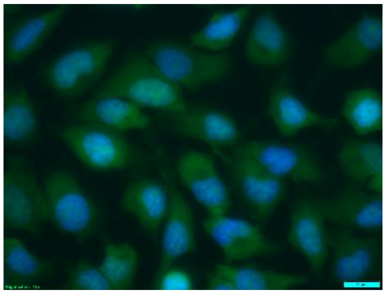

Supplementary Figure 4A. 20 µM Rg3   Each experiment has been repeated twice

**ROS**	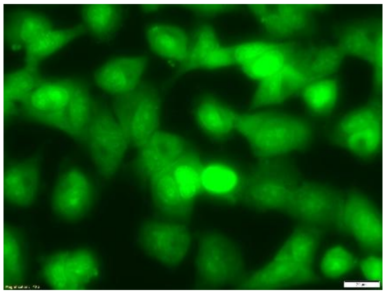	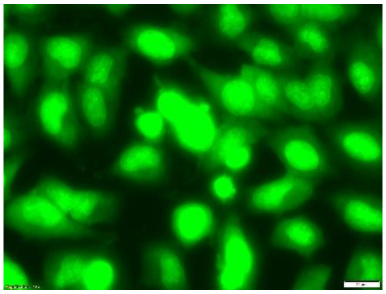
**DAPI**	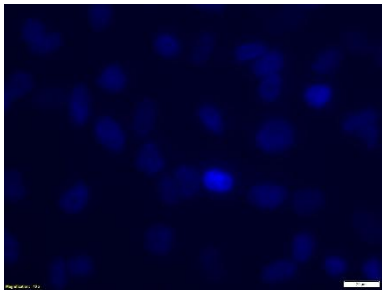	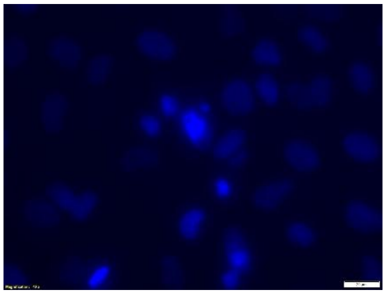
**Merge**	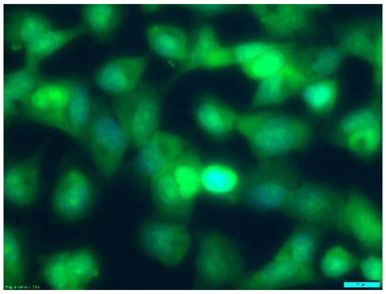	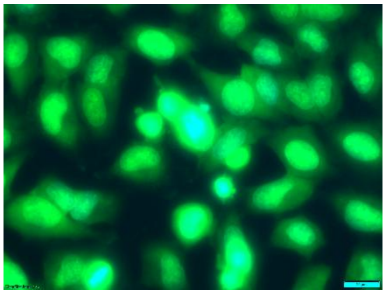

Supplementary Figure 4A. 50 µM Rg3 Each experiment has been repeated twice

**ROS**	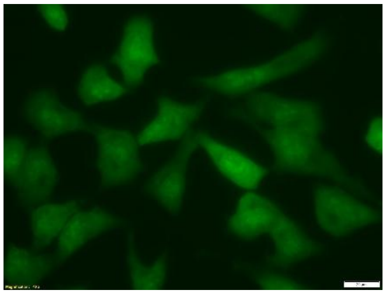	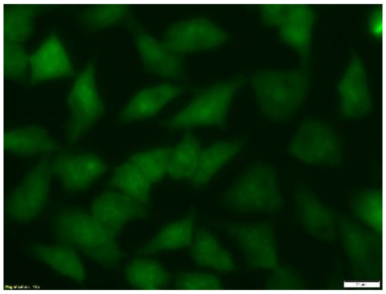
**DAPI**	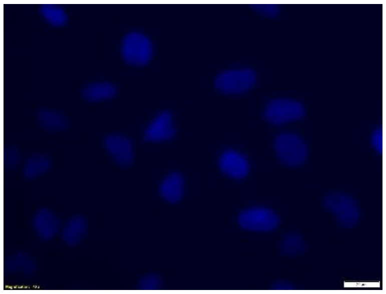	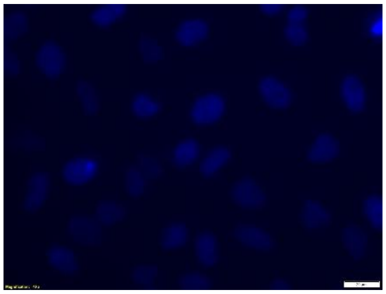
**Merge**	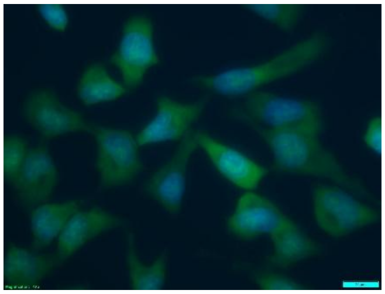	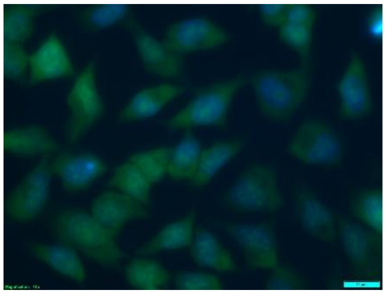

Supplementary Figure 4A. NAC+20 μM Rg3   Each experiment has been repeated twice

Mean signal intensity

TBHP	Control	Rg3 10μM	NAC	Rg3 20μM	Rg3 50μM	NAC+Rg3
158.025100	30.013210	30.013210	25.497290	110.613600	114.172500	57.957930
159.812800	28.379330	35.379330	30.089900	107.516500	108.996000	72.668070
162.510500	26.949070	32.949070	32.050670	117.814900	111.013300	65.743670

Supplementary Figure 4B.

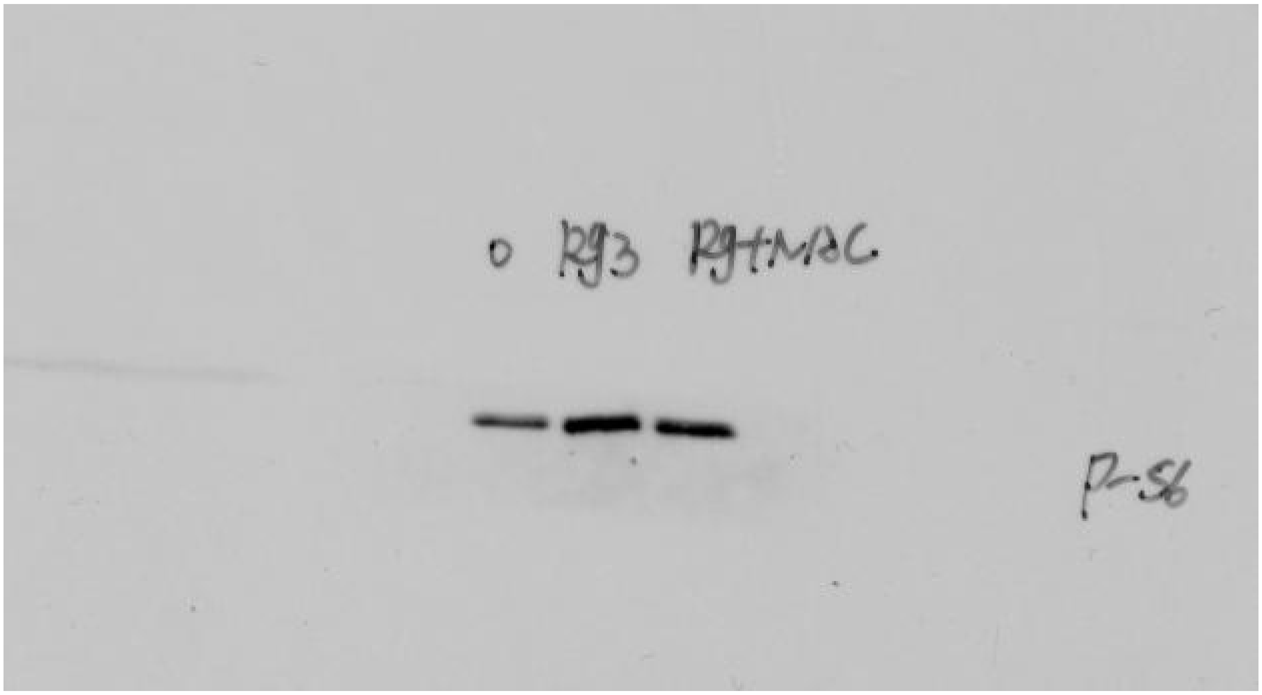

Supplementary Figure4C. P-S6

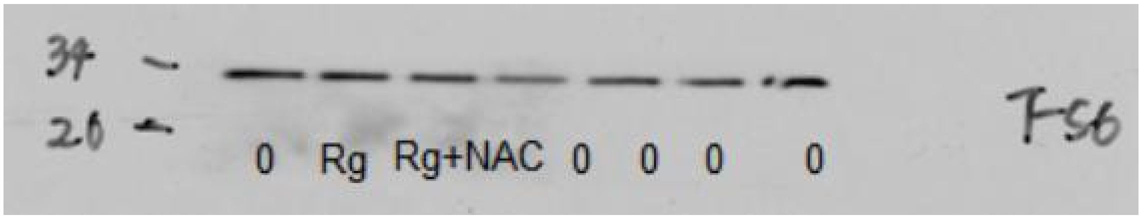

Supplementary Figure 4C. S6

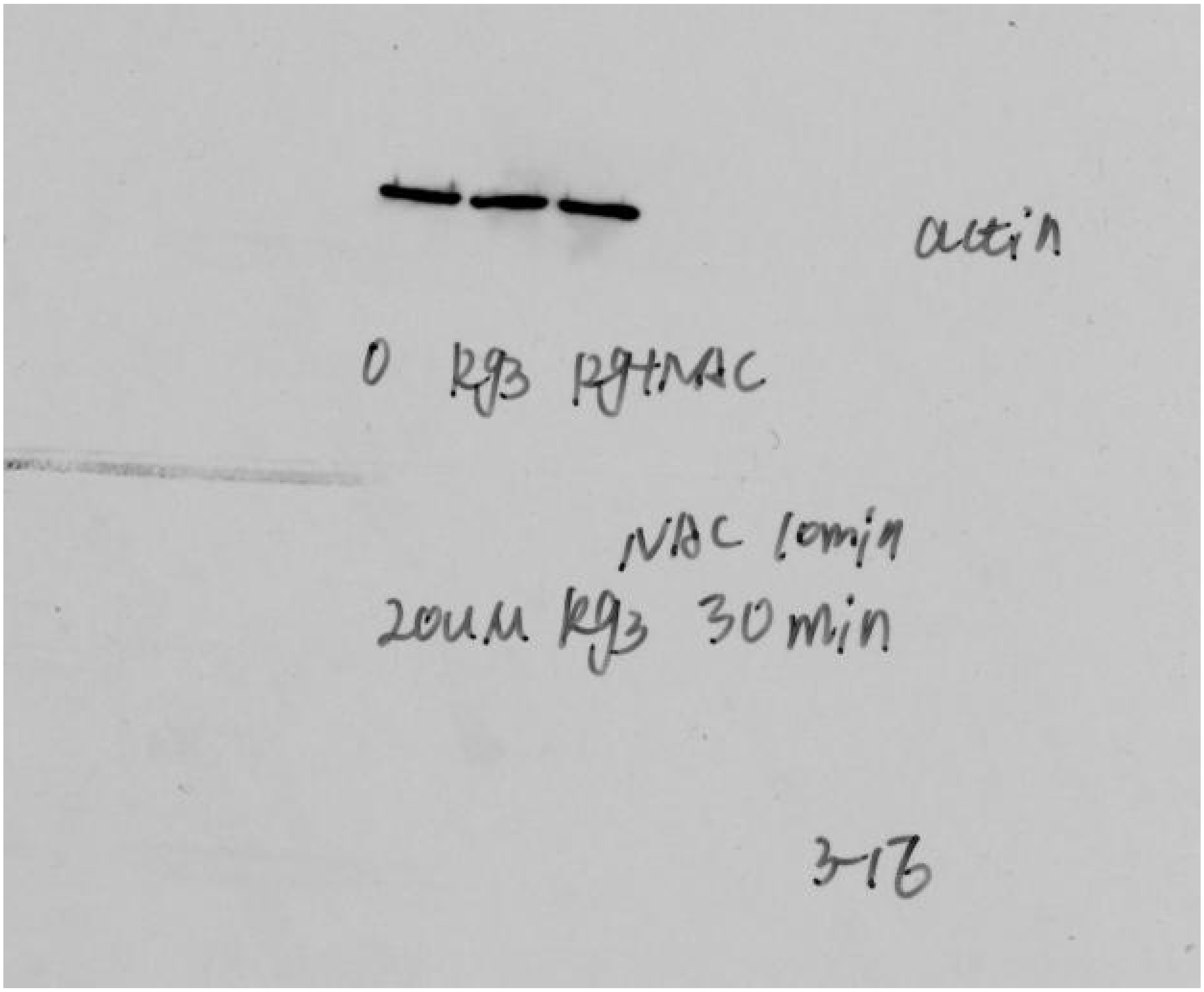

Supplementary Figure 4C. β actin

0μM	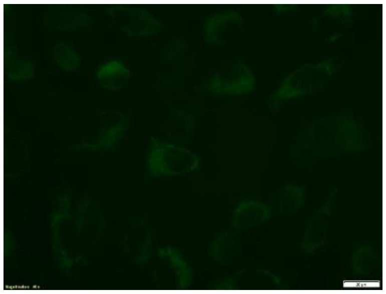	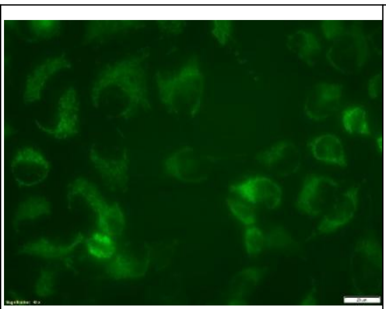	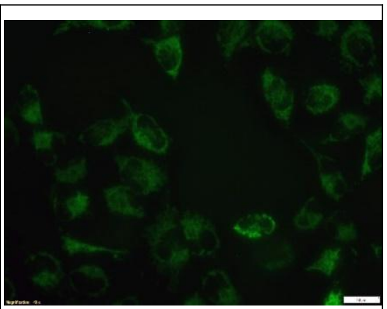
10μM	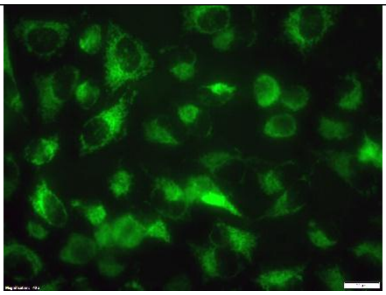	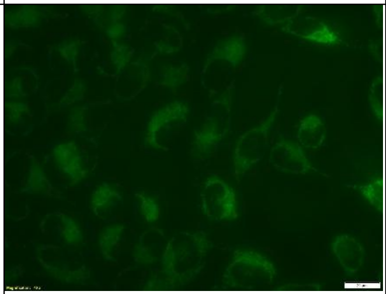	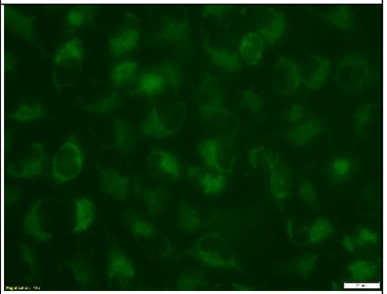
20μM	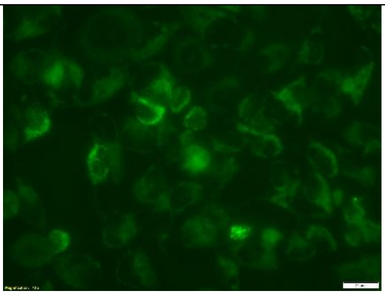	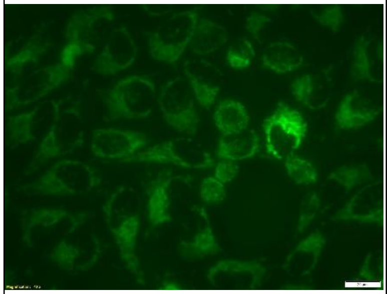	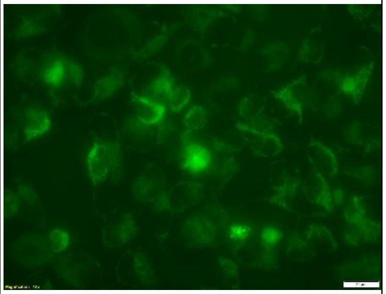
50μM	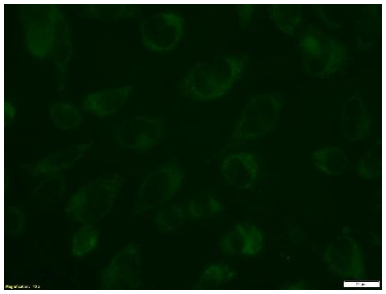	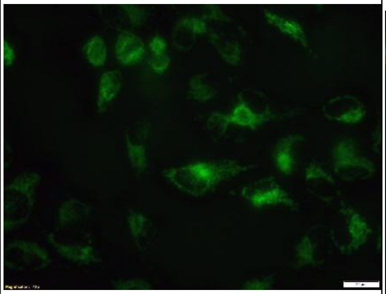	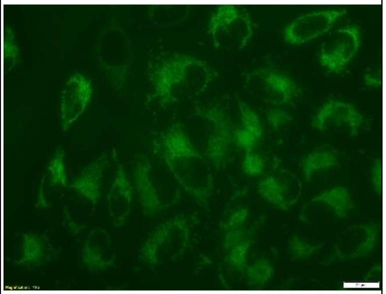

100µM	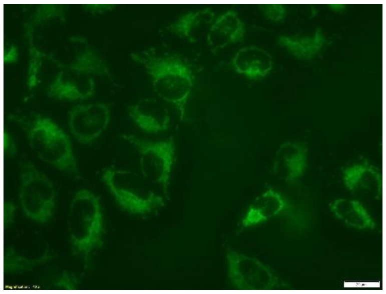	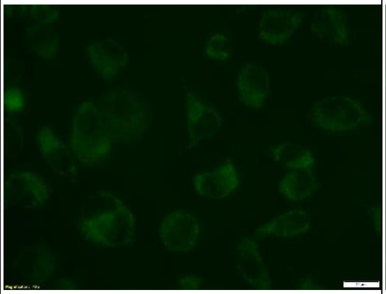	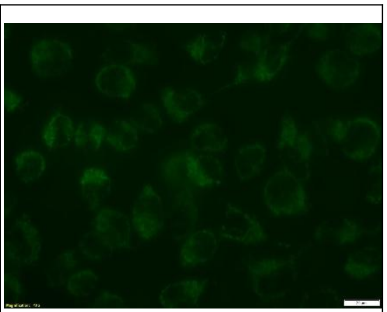

Supplementary Figure 5 A.

Relative of intensity（Green）

0µM Rg3	10µM Rg3	20µM Rg3	50µM Rg3	100µM Rg3
1.	1.505535	1.751514	1.050337	0.9224809
1.	1.561299	1.705529	1.065465	0.9686049
1.	1.529012	1.765167	0.9676824	0.8763568

Supplementary Figure 5 B.

0μM	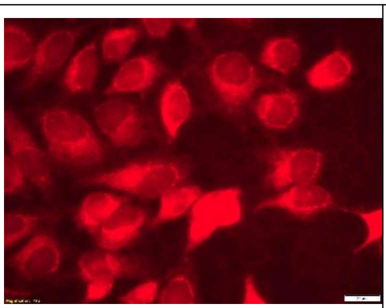	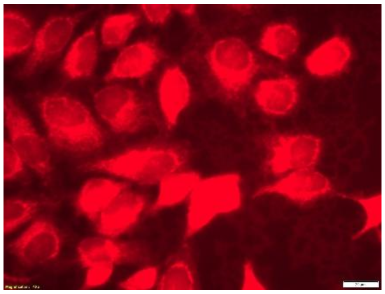	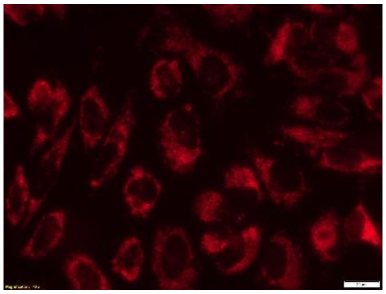
10μM	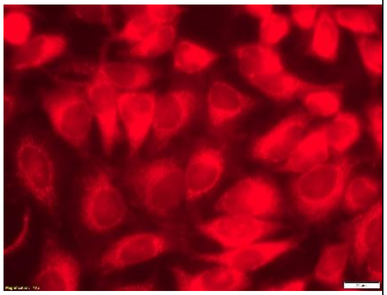	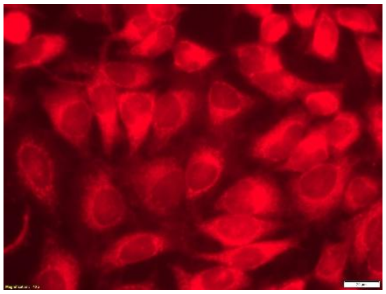	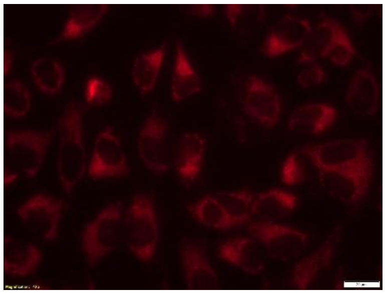
20μM	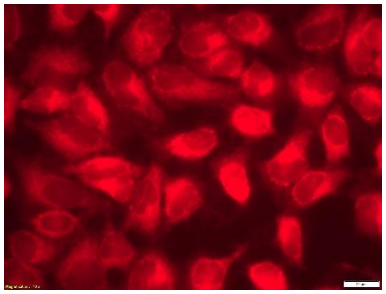	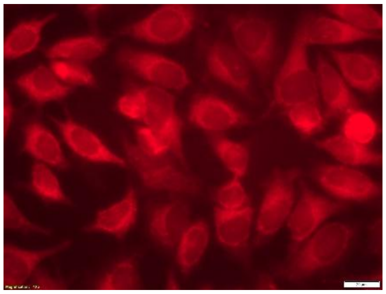	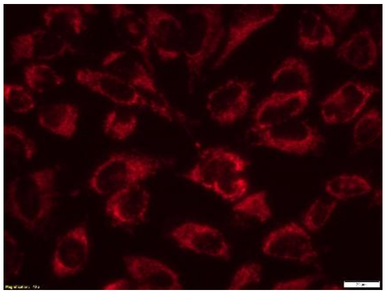
50μM	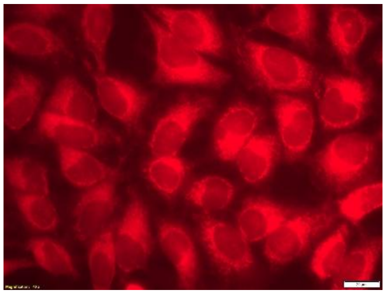	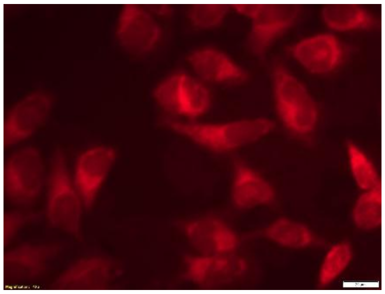	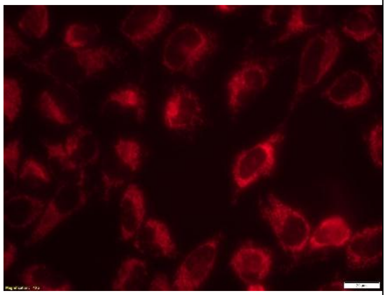

100µM	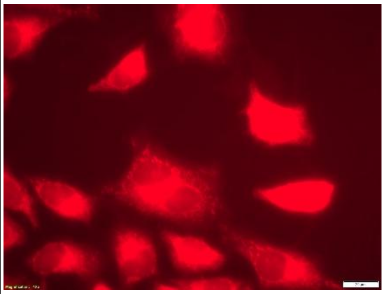	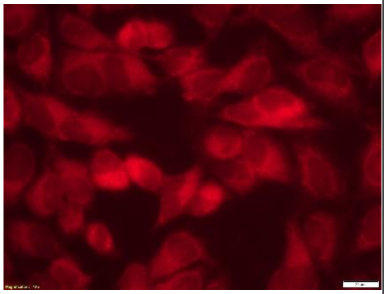	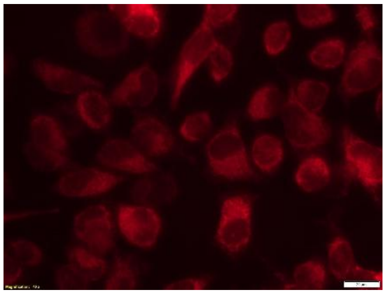

Supplementary Figure 5 C.

Relative of intensity（Red）

0µM Rg3	10µM Rg3	20µM Rg3	50µM Rg3	100µM Rg3
1.	1.011463	1.186534	1.049266	1.187086
1.	1.028955	1.192991	1.014042	0.9629825
1.	1.043867	1.222816	1.028955	1.115357

Supplementary Figure 5 D.

Relative of OCR values

0μM Rg3	10μM Rg3	20μM Rg3
1.	1.355559	1.815725
1.	1.520298	1.675037
1.	1.437928	1.638140

Supplementary Figure 6A.

Relative levels of ATP

0μM Rg3	10μM Rg3	20μM Rg3
1.	1.490598	1.906428
1.	1.375690	1.495600
1.	1.356215	1.344094

Supplementary Figure 6B.
